# Inflammasome activation in infected macrophages drives COVID-19 pathology

**DOI:** 10.1038/s41586-022-04802-1

**Published:** 2022-04-28

**Authors:** Esen Sefik, Rihao Qu, Caroline Junqueira, Eleanna Kaffe, Haris Mirza, Jun Zhao, J. Richard Brewer, Ailin Han, Holly R. Steach, Benjamin Israelow, Holly N. Blackburn, Sofia E. Velazquez, Y. Grace Chen, Stephanie Halene, Akiko Iwasaki, Eric Meffre, Michel Nussenzweig, Judy Lieberman, Craig B. Wilen, Yuval Kluger, Richard A. Flavell

**Affiliations:** 1 Department of Immunobiology, Yale University School of Medicine, New Haven, CT, USA; 2 Department of Pathology, Yale University School of Medicine, New Haven, CT, USA; 3 Program in Cellular and Molecular Medicine, Boston Children’s Hospital, Boston, MA, USA; 4 Department of Pediatrics, Harvard Medical School, Boston, MA, USA; 5 Instituto René Rachou, Fundação Oswaldo Cruz, Belo Horizonte, Minas Gerais, Brazil; 6 Section of Hematology, Yale Cancer Center and Department of Internal Medicine, Yale University School of Medicine, New Haven, CT; 7 Laboratory of Molecular Immunology, The Rockefeller University, New York, NY, USA; 8 Department of Laboratory Medicine, Yale University School of Medicine, New Haven, CT, USA; 9 Howard Hughes Medical Institute, Yale University School of Medicine, New Haven, CT, USA; 10 Howard Hughes Medical Institute, The Rockefeller University, New York, NY, USA; 11 Department of Surgery, Yale University School of Medicine, New Haven, CT, USA; 12 Program of Applied Mathematics, Yale University, New Haven, CT, USA; 13 Computational Biology & Bioinformatics Program, Yale University, New Haven, CT, USA

## Abstract

Severe COVID-19 is characterized by persistent lung inflammation, inflammatory cytokine production, viral RNA, and sustained interferon (IFN) response all of which are recapitulated and required for pathology in the SARS-CoV-2 infected MISTRG6-hACE2 humanized mouse model of COVID-19 with a human immune system^1–20^. Blocking either viral replication with Remdesivir^21–23^ or the downstream IFN stimulated cascade with anti-IFNAR2 *in vivo* in the chronic stages of disease attenuated the overactive immune-inflammatory response, especially inflammatory macrophages. Here, we show SARS-CoV-2 infection and replication in lung-resident human macrophages is a critical driver of disease. In response to infection mediated by CD16 and ACE2 receptors, human macrophages activate inflammasomes, release IL-1 and IL-18 and undergo pyroptosis thereby contributing to the hyperinflammatory state of the lungs. Inflammasome activation and its accompanying inflammatory response is necessary for lung inflammation, as inhibition of the NLRP3 inflammasome pathway reverses chronic lung pathology. Remarkably, this same blockade of inflammasome activation leads to the release of infectious virus by the infected macrophages. Thus, inflammasomes oppose host infection by SARS-CoV-2 by production of inflammatory cytokines and suicide by pyroptosis to prevent a productive viral cycle.

## Introduction:

Acute SARS-CoV-2 infection resolves in most patients but becomes chronic and sometimes deadly in about 10–20%^1–7,14–16,20,24–27^. Two hallmarks of severe COVID-19 are a sustained interferon (IFN) response and viral RNA persisting for months^1–18,20,24–28^. This chronicity is recapitulated in SARS-CoV-2 infected MISTRG6-hACE2 humanized mice^19^. Copious interleukin (IL)-1β, IL-18 and lactate dehydrogenase (LDH) correlate with COVID-19 severity in patients, suggesting a role for inflammasome activation and pyroptosis in pathology^5–7,14–18,29^. Here, we show that human lung macrophages are infected by SARS-CoV-2. Replicating SARS-CoV-2 in these human macrophages activates inflammasomes and initiates an inflammatory cascade with a unique transcriptome, results in pyroptosis, and contributes to the downstream type-I-IFN response. Blocking either viral replication, the downstream IFN response or inflammasome activation *in vivo* during the chronic phase of the disease attenuates many aspects of the overactive immune-inflammatory response, especially the inflammatory macrophage response, and disease.

## Results:

### Viral replication and the IFN response

**Extended data figure 1.**
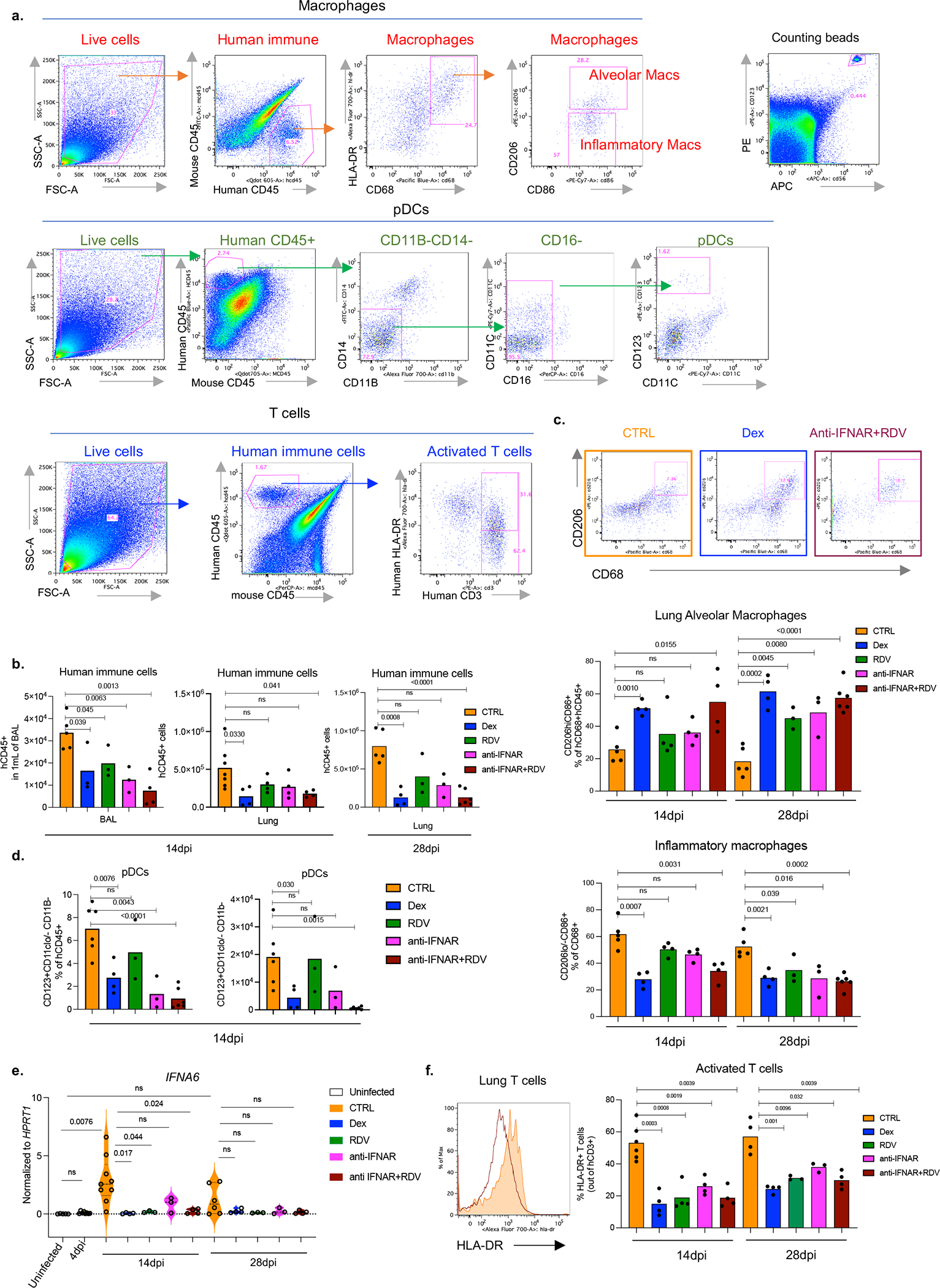
Targeting viral replication and the downstream interferon response attenuates the hyperactive immune/inflammatory response (matched to figure 1). a. Representative gating strategy of human immune cells in the lungs of SARS-CoV-2 infected MISTRG6-hACE2 mice. Cells isolated from lungs or bronchioalveolar lavage (BAL) were stained with antibodies against human CD45, HLA-DR, CD68, CD16, CD14, CD206, CD86, CD11B, CD11C, CD123, CD3, and mouse CD45. Cell numbers were calculated using counting beads. b. Human immune cells (numbers) in BAL (14dpi) or lungs (14 and 28dpi) of SARS-Cov-2 infected MISTRG6-hACE2 mice treated with dexamethasone (Dex), Remdesivir (RDV), anti-IFNAR2 or a combined therapy of Remdesivir (RDV) and anti-IFNAR2. 14dpi, BAL: CTRL-infected n=5, Dex; RDV anti-IFNAR2 n=3; anti-IFNAR2+RDV n=4 biologically independent mice examined over 2 independent experiments. 14dpi, Lung: CTRL-infected n=7; Dex, RDV, anti-IFNAR2, anti-IFNAR2+RDV n=4 biologically independent mice examined over 3 independent experiments. 28dpi, lung: CTRL infected n=5, Dex n=4, RDV n=3, anti-IFNAR2 n=3, anti-IFNAR2+RDV n=6 biologically independent mice examined over 3 independent experiments. Means with individual datapoints plotted. Unpaired, two-tailed t-test. P<0.0001=8.19×10^−5^. c. Representative flow cytometry plots and frequencies of alveolar macrophages (AMs) (middle: hCD206^hi^hCD86^+^hCD68^+^) or inflammatory macrophages (bottom: hCD206^lo/−^hCD86^+^hCD68^+^) in 14dpi or 28dpi lungs of treated or untreated MISTRG6-hACE2 mice. 14dpi: CTRL infected n=5, Dex n=4, RDV n=4, anti-IFNAR2 n=4, anti-IFNAR2+RDV n=4 biologically independent mice examined over at least 2 independent experiments. 28dpi: CTRL infected n=5, Dex n=4, RDV n=3, anti-IFNAR2 n=3, anti-IFNAR2+RDV n=6 biologically independent mice examined over 3 independent experiments. Means with individual datapoints. Unpaired, two-tailed t-test. P<0.0001=4.67×10^−5^. d. Frequencies (left) and numbers (right) of lung pDCs at 14dpi. CTRL-infected n=6, Dex n=4, RDV, anti-IFNAR2 n=3, anti-IFNAR2+RDV n=6 mice examined over at least 2 experiments. Means with datapoints. Unpaired, two-tailed t-test. P<0.0001=7.29×10^−5^. e. *IFNA* transcript levels measured by qPCR in treated or control untreated MISTRG6-hACE2 mice infected with SARS-CoV-2: Uninfected n=5; CTRL infected: 4dpi n=8, 14dpi n =9, 28dpi n=: 6; Dex 14dpi n=4, Dex 28dpi= 4; RDV 14 and 28dpi n=3, anti-IFNAR2 14 and 28dpi n=3, anti-IFNAR2+ Remdesivir 14 and 28dpi n=4 biologically independent mice examined over at least 2 independent experiments. Normalized to *HPRT1*. Violin plots with individual datapoints. Unpaired, two-tailed t test. f. Representative histograms and frequencies of HLA-DR^+^ activated T cells in treated or control mice. 14dpi: CTRL-infected n=5, Dex, RDV, anti-IFNAR2, anti-IFNAR2+RDV n=4; 28dpi: CTRL infected, Dex, anti-IFNAR2+RDV n=4, RDV, anti-IFNAR2 n=3 biologically independent mice examined over 3 independent experiments. Means with datapoints. Unpaired, two-tailed t-test. Some of the data associated with dexamethasone therapy used here as a control have been reported^19^.

**Figure 1.**
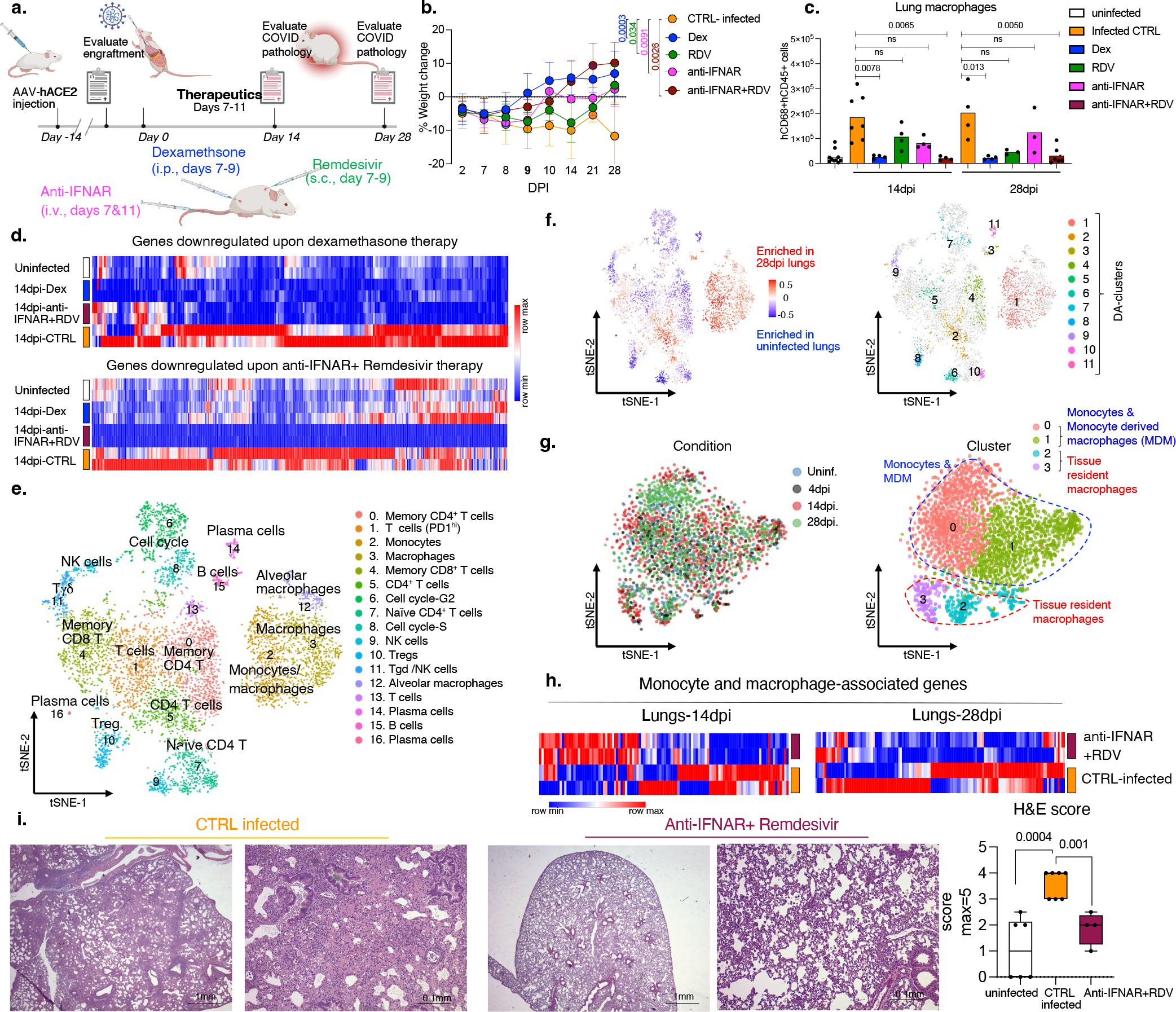
Targeting viral replication and downstream interferon signaling ameliorates chronic COVID-19. a. Therapy Schematic: SARS-CoV-2 infected MISTRG6-hACE2 mice were treated with Dexamethasone(dex) and Remdesivir(RDV) at 7,8,9 dpi, with anti-IFNAR2 at 7, 11dpi and analyzed at 14 or 28dpi. b. Post-infection weight changes. 28dpi: CTRL-infected n=5, Dex, anti-IFNAR2+RDV n=4, RDV, anti-IFNAR2 n=3 mice examined over at least 2 experiments. Means with SD. Unpaired, two-tailed t-test. c. Human macrophages in lungs. Uninfected: n=10; 14dpi: CTRL-infected n=7 Dex, RDV, anti-IFNAR, anti-IFNAR+RDV n=4; 28dpi: CTRL-infected, Dex n=4, RDV, anti-IFNAR n=3, anti-IFNAR+RDV n=6 mice examined over 3 experiments. Means with datapoints. Unpaired, two-tailed t-test. d. Heatmap of genes suppressed by therapy in lungs (Log2, Foldchange >1; P-adj with the Bonferroni correction <0.05). Differential expression by DESeq2. Statistics by Wald-test. Transformed (min-max), normalized counts of duplicates. Hierarchical clustering (one-minus Pearson). e. t-distributed stochastic neighbor embedding (*t*-SNE) plot of human immune cells from uninfected or infected lungs (28dpi). Pooled duplicates. Cluster marker genes identified with Wilcoxon-test (Extended data Fig. 2e). Uninfected=3,655, 28dpi=3,776 cells analyzed. f. t-SNE plots highlighting differentially abundant (DA) human immune cell populations identified by DA-seq^76^. Top: Distribution/enrichment of DA-populations. Bottom: DA-clusters. g. *t*-SNE plots of human monocyte/macrophage clusters from 4dpi, 14dpi and 28dpi and uninfected lungs. Left: dpi, right: clusters. Different conditions integrated as described in methods^75^. Marker genes identified with Wilcoxon-test (Extended data Fig.3a,b). P-adj with the Bonferroni correction. Uninfected=438, 4dpi=336, 14dpi=793, 28dpi=1368 cells analyzed. h. Heatmap visualizing response to the combined therapy based on DEGs associated with monocytes and macrophages. Transformed (min-max), normalized expression of duplicates. Hierarchical clustering (one-minus Pearson). i. Representative H&E staining and box plot of histopathological scores. Uninfected n=6, CTRL-infected n=7, anti-IFNAR2+RDV n=4 mice examined over 3 experiments. Whiskers: smallest/minimum to the largest/maximum value. Box: 25^th^-75^th^ percentiles. Center line: median. Unpaired, two-tailed, t-test. Data associated with dexamethasone used here as a control have been reported^19^.

Chronic interferon is associated with disease severity and impaired recovery in influenza infection^30^. To test whether a viral RNA-dependent type-I IFN response was a driver of chronic disease, we treated SARS-CoV-2 infected MISTRG6-hACE2 mice with Remdesivir^21–23^ and/or anti-IFNAR2 antibody (Fig. 1a) to inhibit viral replication and the IFN-response downstream of chronic infection, respectively. As control, we used dexamethasone, which reverses many aspects of immunopathology in infected MISTRG6-hACE2 mice^19^ and in patients^31^. Although Remdesivir and anti-IFNAR2 alone were partially therapeutic, combined therapy achieved more rapid weight recovery and suppression of the immune inflammatory response, especially macrophages, as effectively as dexamethasone (Fig. 1b–c, Extended data Fig. 1a–f), suggesting a combinatorial effect of Remdesivir and anti-IFNAR2 in chronic infection.

**Extended data figure 2.**
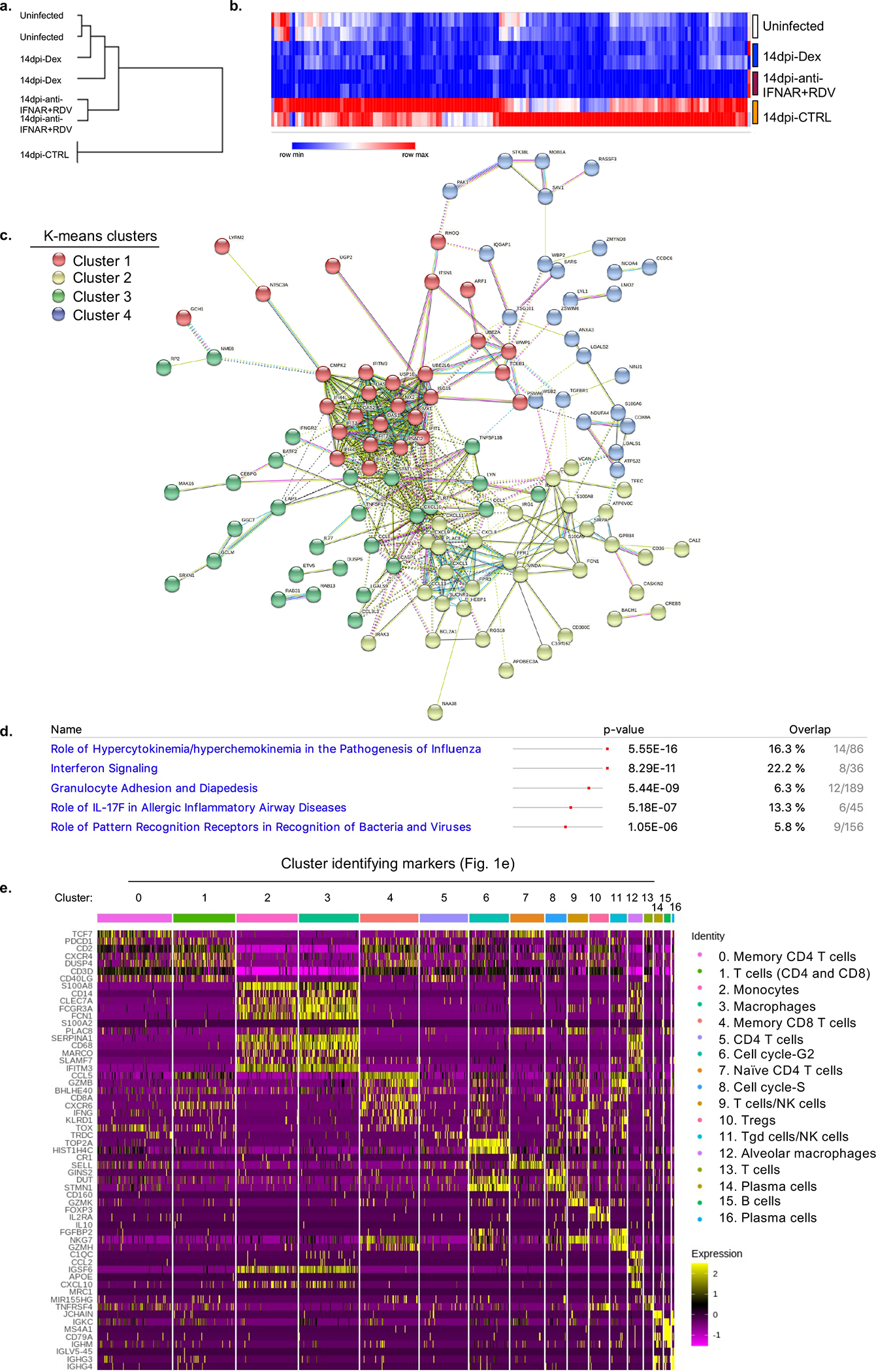
Anti-IFNAR2 and Remdesivir therapy reverses infection induced transcriptional changes (matched to figure 1). a. Similarity comparison of uninfected, infected, and therapeutically manipulated lungs based on dexamethasone suppressed genes. Pearson correlation. Duplicates analyzed for each condition. b. Genes suppressed by both dexamethasone and combined therapy of Remdesivir and anti-IFNAR2 (Log2, Foldchange <−1, P adj<0.05). P adj: For the adjusted P values the Bonferroni correction was used. Duplicates analyzed for each condition. Dexamethasone suppressed genes significantly overlapped with genes significantly suppressed by combined anti-IFNAR2 and Remdesivir therapy (64% overlap). See Table S1 for a full list of genes and their normalized expression. c. Network analysis (STRING v11.0) of genes suppressed by both dexamethasone and combined therapy of Remdesivir and anti-IFNAR2 (as shown in Extended data figure 2b). Duplicates analyzed for each condition. K- means clustering (n=4). d. Pathway (Ingenuity) analysis of genes suppressed by both dexamethasone and combined therapy of Remdesivir and anti-IFNAR2 (as shown in Extended data Fig. 2b). Duplicates analyzed for each condition. Fisher’s Exact Test was used to determine statistical significance in the overlap between the dataset genes and the genes suppressed by therapy. e. Transcriptional landscape of human immune cells at single cell level in uninfected or infected (28dpi). MISTRG6-hACE2 mice. Cluster identifying genes comparing human immune cells from infected (28dpi) or uninfected lungs for 17 clusters shown in Fig 1e. Marker genes for each cluster of cells were identified using the Wilcoxon test with Seurat. Pooled duplicates analyzed for each condition.

**Extended data figure 3.**
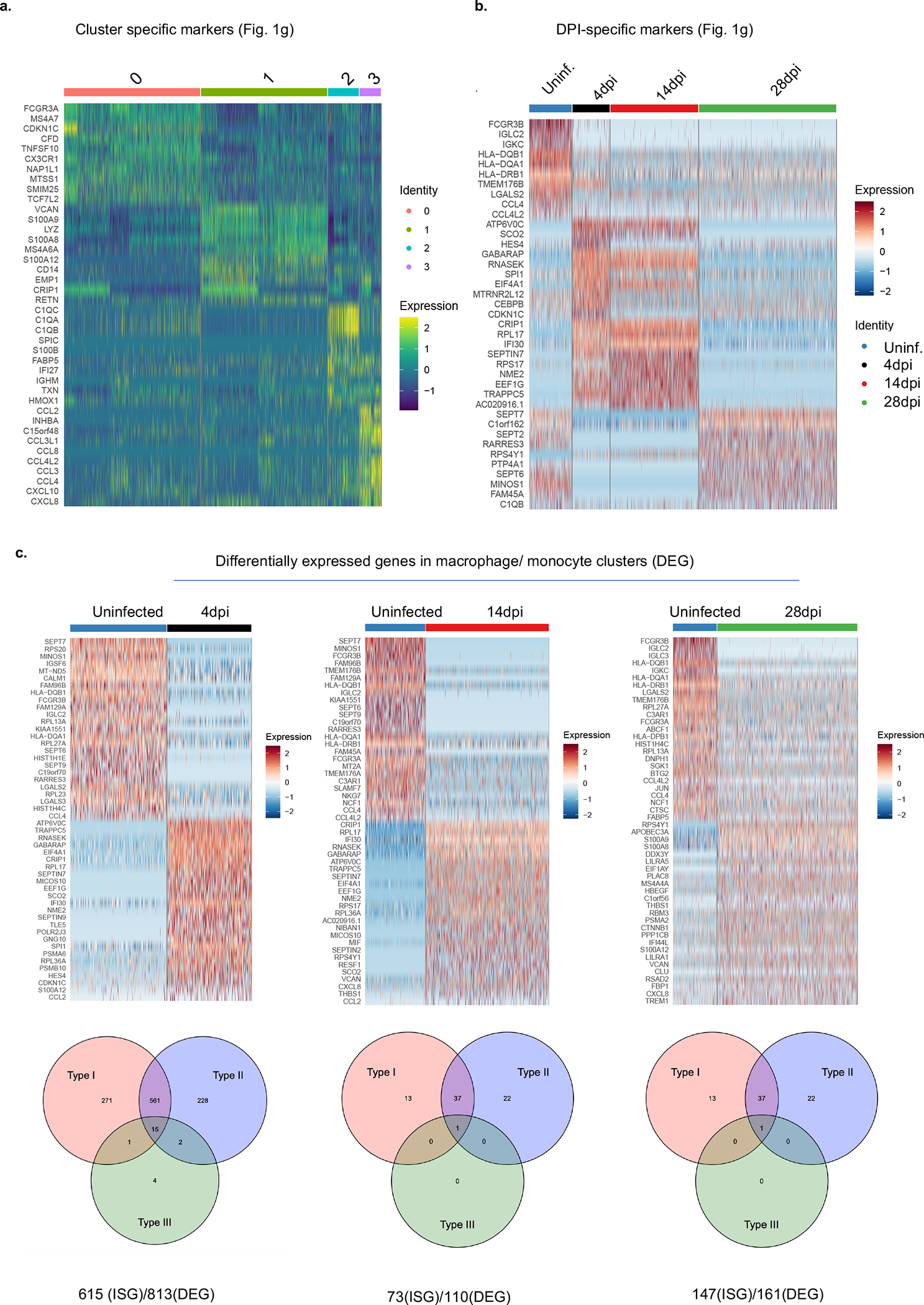
Deeper characterization of monocyte/macrophage clusters at early (4dpi) or late (14 and 28dpi) SARS-CoV-2 infection(matched to figure 1). a. Heatmap visualizing cluster identifying genes comparing human monocytes and macrophages from infected (4, 14 or 28dpi) or uninfected lungs (as shown in Fig 1g). Pooled duplicates. Uninfected: 438 cells, 4dpi: 336 cells, 14dpi: 793 cells, 28dpi: 1368 cells were analyzed. This analysis allowed step by step characterization of the inflammatory macrophage response. Marker genes for each cluster of cells were identified using the Wilcoxon test (two-tailed) with Seurat. b. Temporal distribution of transcriptional changes associated with monocytes and macrophages in infected (4, 14 or 28dpi) or uninfected lungs (as shown in Fig 1g). Pooled duplicates analyzed. Uninfected: 438 cells, 4dpi: 336 cells, 14dpi: 793 cells, 28dpi: 1368 cells included in analysis. c. Top: Heatmap of representative genes that are differentially regulated (DEGs) in human macrophages from 4, 14, 28dpi lungs compared with uninfected lungs. Uninfected: 438 cells, 4dpi: 336 cells, 14dpi: 793 cells, 28dpi: 1368 cells included in analysis. Bottom: Distribution of interferon stimulated genes within these DEGs. Pooled duplicates analyzed.

**Extended data figure 4.**
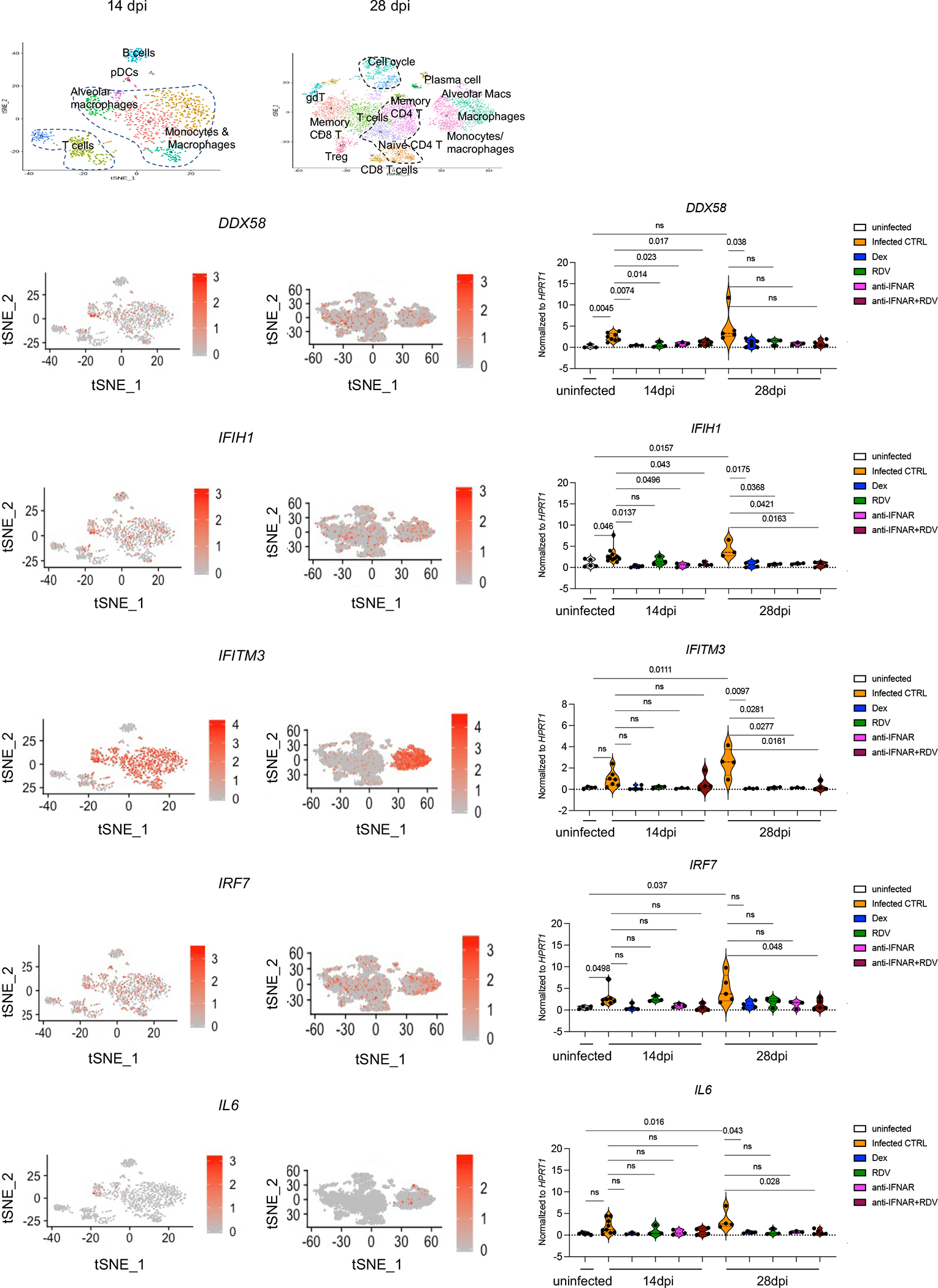
Therapeutics reduced expression of a representative list of interferon stimulated genes- ISGs (*DDX58, IFIH1, IFITM3, IRF7*) or inflammatory markers (*IL6*) (matched to figure 1). Relative expression of interferon inducible or inflammatory genes in treated or untreated MISTRG6- hACE2 mice infected with SARS-CoV-2 mice at 14dpi or 28dpi. Uninfected baseline expression values are presented as reference. The distribution of cells that preferentially express these genes is overlayed on the tSNE plots showing 14dpi and 28dpi human immune cells. *IFITM3* and *IL6* were particularly enriched in macrophage/monocyte clusters, while *IRF7*, *DDX58* and *IFIH1* were enriched in multiple immune cells such as T cells, B cells, and myeloid cells. Normalized to *HPRT1*. *DDX58*: uninfected n=3; CTRL-infected: 14dpi n=8, 28dpi n=: 5; Dex 14dpi n=3, 28dpi n= 6; RDV 14 and 28dpi n=3; anti-IFNAR2 14 and 28 dpi n=3, anti-IFNAR2+ Remdesivir 14 and 28dpi n=5 biologically independent mice examined over at least 2 independent experiments. *IFIH1*: uninfected n=5; CTRL-infected: 14dpi n =11, 28dpi n=: 3; Dex 14 and 28dpi n=4; RDV 14 and 28dpi n=3; anti-IFNAR2 14 and 28 dpi n=3, anti-IFNAR2+ Remdesivir 14 dpi n=4 and 28dpi n=5 biologically independent mice examined over at least 2 independent experiments. *IFITM3*: uninfected n=4; CTRL-infected: 14dpi n =7, 28dpi n=: 4; Dex 14 and 28dpi n=4; RDV 14 and 28dpi n=3; anti-IFNAR2 14 and 28 dpi n=3, anti-IFNAR2+ Remdesivir 14 and 28dpi n=4 biologically independent mice examined over at least 2 independent experiments. *IRF7*: uninfected n=4; CTRL-infected: 14dpi n =7, 28dpi n=: 5; Dex 14 and 28dpi n=4; RDV 14 and 28dpi n=3; anti-IFNAR2 14 and 28 dpi n=3, anti-IFNAR2+ Remdesivir 14dpi n=4, 28dpi n=5 biologically independent mice examined over at least 2 independent experiments. *IL6*: uninfected n=5; CTRL-infected: 14dpi n =9, 28dpi n=: 4; Dex 14dpi n=3, 28dpi n=4; RDV 14 and 28dpi n=3; anti-IFNAR2 14 and 28 dpi n=3, anti- IFNAR2+ Remdesivir 14dpi n=4, 28dpi n=5 biologically independent mice examined over at least 2 independent experiments. Unpaired, two-tailed t-test.

**Extended data figure 5.**
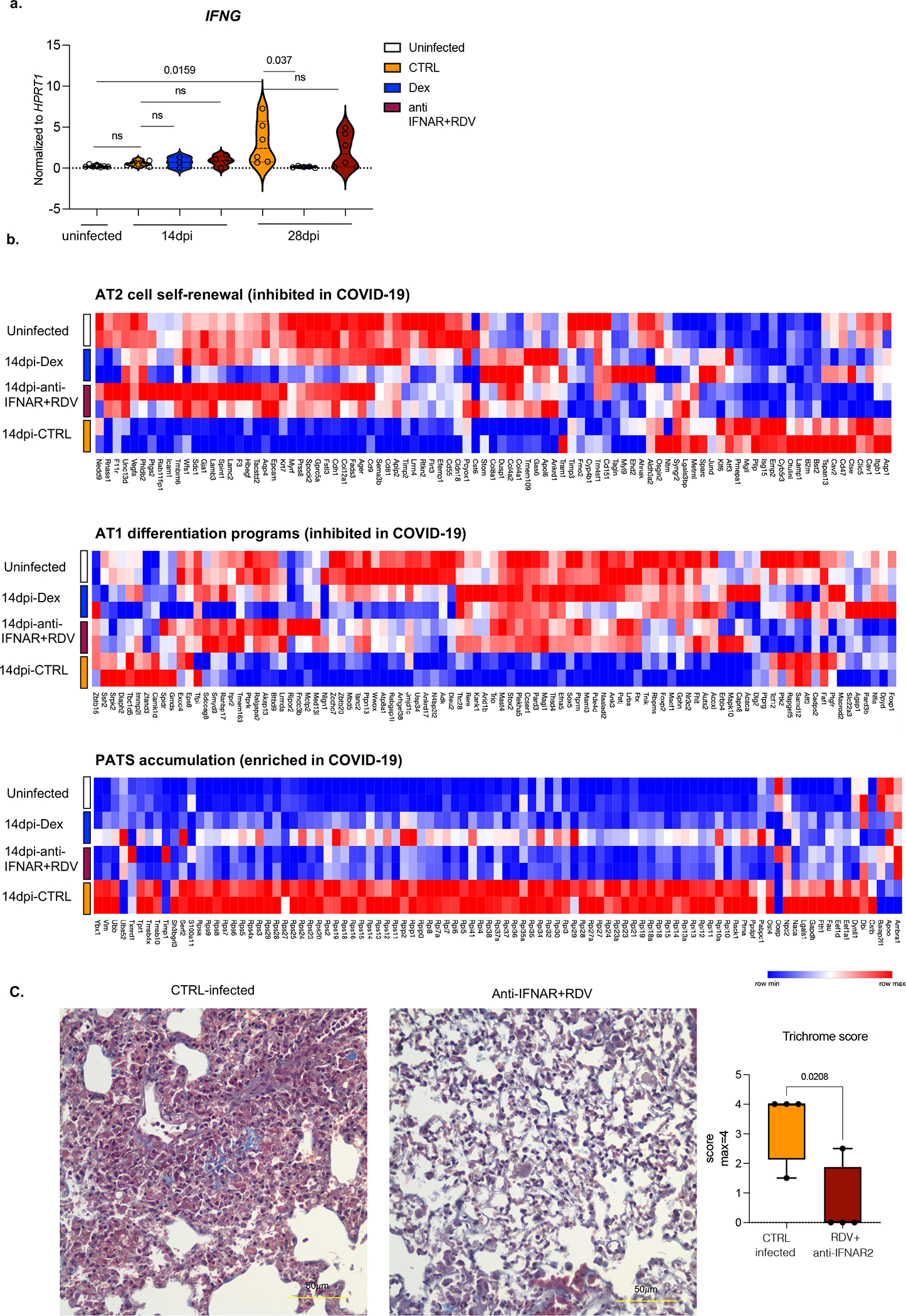
Anti-IFNAR2 and Remdesivir combined therapy reverses fibrotic transcriptional signature and prevents the transition to fibrosis seen in the infected mice (matched to figure 1). a. Relative expression of *IFNG* in treated or untreated MISTRG6-hACE2 mice infected with SARS-CoV-2 mice at 14dpi or 28dpi. Uninfected baseline expression values are presented as reference. Normalized to *HPRT1*. Uninfected n=7; CTRL infected: 14dpi n =7, 28dpi n=: 6; Dex 14dpi=3, 28dpi= 5; anti-IFNAR2+ Remdesivir 14dpi n=4, 28dpi n=6. over at least 2 independent experiments. Unpaired, two-tailed t-test. b. Heatmap of AT2 cell self-renewal and AT1 differentiation and pre-alveolar type 1 transitional cell state (PATS) associated genes at in uninfected or infected (14dpi) lungs in response to therapeutics. AT2 cell self-renewal and AT1 differentiation gene signature was inhibited while PATS gene signature was enriched in autopsy lungs of patients with severe COVID-19^7^. Top differentially expressed genes in epithelial cluster 7 of autopsy lungs^7^ were used in the analysis. Duplicates were analyzed for each condition. Normalized counts of duplicates visualized as min-max transformed values, calculated by subtracting row mean and diving by SD for each gene. Rows (genes) clustered by hierarchical clustering (one-minus Pearson). c. Representative images of Trichrome staining and box and whisker plot (min to max, with all datapoints) of the trichrome scoring of MISTRG6-hACE2 mice treated with a combined therapy of Remdesivir and anti-IFNAR2 or not (CTRL infected). The whiskers go down to the smallest value (minimum) and up to the largest value (maximum). The box extends from the 25th to 75th percentiles. The median is shown as a line in the center of the box. N=4 biologically independent mice examined over 2 independent experiments. Unpaired, two-tailed t-test.

We assessed the impact of therapeutics on the lung transcriptome. Both dexamethasone and the combined therapy reversed overactive immune transcripts to uninfected animal levels (Fig. 1d, Extended data Fig. 2a, b Table S1). The reduced transcripts were enriched for chemokine and cytokine networks (*CXCL10, CXCL8, CCL2*), inflammatory (*TLR7, NLRP3, CASP1*) and anti-viral (*MPO, OAS1, OAS2*) response, and interferon stimulated genes (ISGs) (*IFITM3, IFITM2, IRF7*) (Table S1, Extended data Fig. 2c, d), emphasizing the central role of IFN signaling and inflammatory cytokine-chemokines in chronic COVID-19. Comparison of single-cell transcriptomes of human immune cells from infected mice with their uninfected counterparts (Fig. 1e,–g, Extended data Fig. 2e) showed tissue-resident macrophages, such as alveolar macrophages (AMs), activated at the peak of infection, followed by an inflammatory response with infiltrating monocytes and monocyte-derived macrophages (Fig. 1g, Extended data figure 3a–c, Table S2). As macrophages differentiated, they maintained their inflammatory signature and activated status throughout infection (Extended data Fig.3a–c, Table S2). All macrophage subsets were enriched for ISGs at all timepoints (Extended data Fig. 3c). These ISGs were suppressed upon anti-IFNAR2/Remdesivir combination therapy (Fig. 1h, Extended data Fig. 4, Table S3). Yet, key anti-viral responses such as *IFNG* primarily produced by cytotoxic T cells were spared (Extended data Fig. 5a), highlighting the selective effects of combined anti-IFNAR2/Remdesivir therapy on chronic COVID-19 pathology. Consistent with the fibrosis, seen both in patients^32–36^ and humanized mice^19^, alveolar self-renewal and differentiation programs were inhibited, resulting in the accumulation of pre-alveolar type 1 transitional cell state (PATS) program in pneumocytes^7,37–39^ that was reversed in infected MISTRG6-hACE2 mice by anti-IFNAR2/Remdesivir combination therapy, restoring self-renewal and differentiation programs (Extended data Fig. 5b). Overall, reducing chronic inflammation enhanced lung tissue recovery, and prevented transition to fibrosis seen in humanized mice^19^ (and humans^32–36^) (Fig. 1i, Extended data Fig. 5c).

### SARSCoV2 replicates in human macrophages

**Extended data figure 6.**
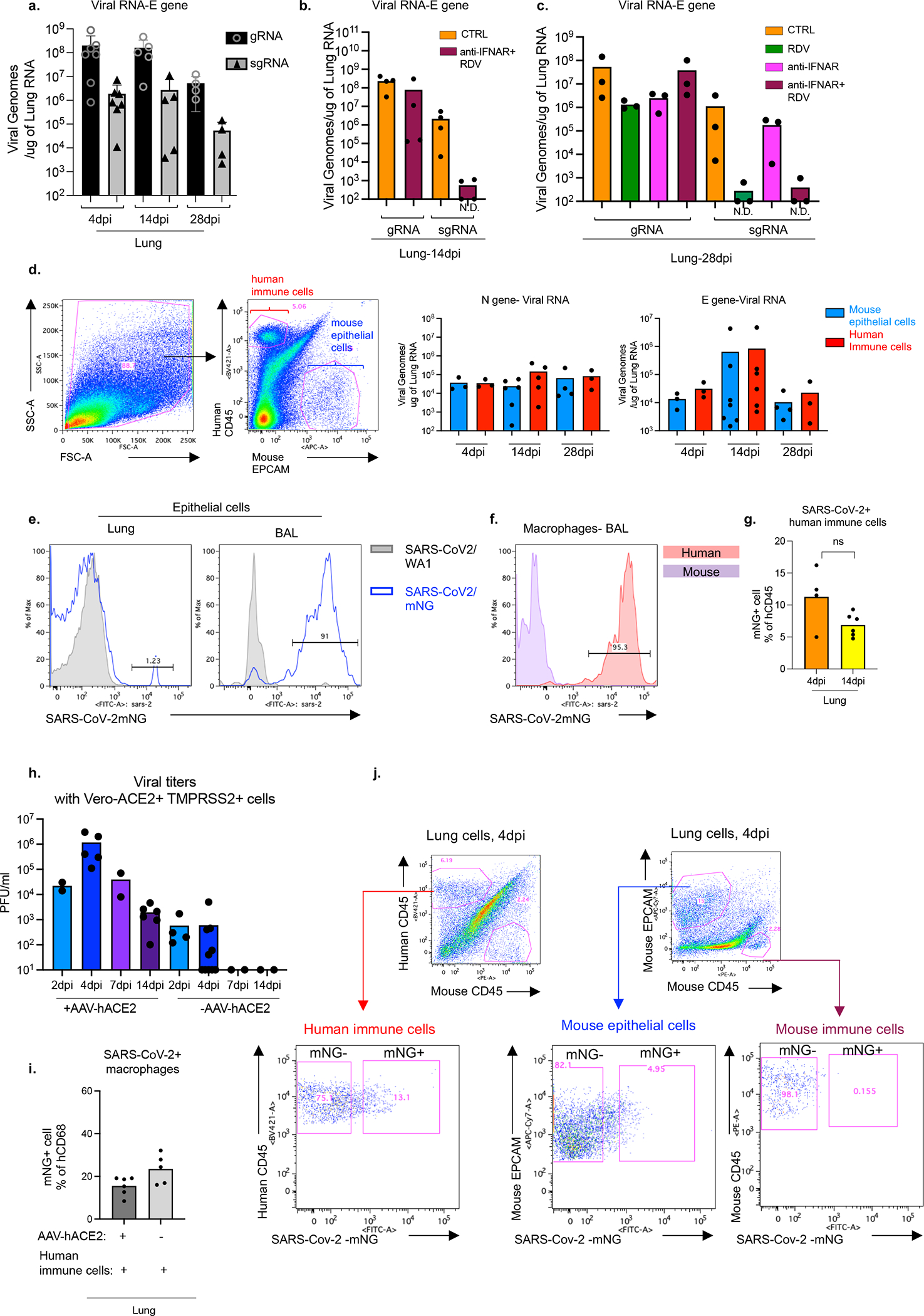
Cellular source of persistent SARS-CoV-2 viral RNA and sustained viral replication in lungs (matched to figure 2). a. Quantification of genomic (gRNA) and subgenomic (sgRNA) viral RNA (E-gene) in whole homogenized lung tissue at 4, 14 and 28dpi. 4dpi: n=7, 14dpi n=5, 28dpi n=4 biologically independent mice examined over 3 independent experiments. Means with all datapoints and SD. b. Quantification of genomic (gRNA) and subgenomic (sgRNA) viral RNA (E-gene) in whole homogenized lung tissue at 14dpi in mice treated with combined therapy of Remdesivir and anti-IFNAR2. CTRL: n=4, anti-IFNAR2+RDV: n=4 biologically independent mice examined over 2 independent experiments. N.D.=not detected. c. Quantification of genomic (gRNA) and subgenomic (sgRNA) viral RNA (E-gene) in whole homogenized lung tissue at 28dpi in mice treated with Remdesivir, anti-IFNAR2 or combined therapy of Remdesivir and anti-IFNAR2. N=3 biologically independent mice representative of 2 independent experiments. N.D.=not detected. d. Representative gating strategy for sorting human immune cells (human CD45+) or epithelial cells (mouse EPCAM+) from lungs of mice infected with SARS-CoV-2 and quantification of viral RNA (E and N genes) in these sorted cells. N gene: 4dpi n=3, 14dpi n=6(epithelial), n=5 (immune), 28dpi n=4 (epithelial) n=3 (immune) biologically independent mice analyzed over 3 independent experiments. E gene: 4dpi n=3, 14dpi n=7 (epithelial), n=6 (immune), 28dpi n=4 (epithelial) n=3 (immune) biologically independent mice analyzed over 3 independent experiments. e. mNG signal in epithelial (EPCAM+) cells from lungs and BAL of mice infected with reporter SARS-CoV-2-mNG or control wild type SARS-CoV-2/WA1. mNG is expressed in infected cells following viral replication. Representative of n=4 biologically independent mice examined over 2 independent experiments. f. Representative histograms of mNG expression in human or mouse lung macrophages isolated from BAL of infected MISTRG6-hACE2 mice at 4dpi. Representative of n=3 biologically independent mice examined over 2 independent experiments. g. Frequencies of mNG+ cells within human lung immune cells (hCD45^+^) of SARS-CoV-2-mNG infected MISTRG6-ACE2 mice at 4dpi and 14dpi. 4dpi n=4, 14dpi n=6 biologically independent mice examined over at least 2 experiments. Unpaired, two-tailed t-test. P value=0.066. h. Viral titers measured as PFU using Vero ACE2+ TMPRSS2+ cells that over express ACE2 from lung homogenates of MISTRG6 mice transduced with AAV-hACE2 (+AAV) or not (−AAV) and infected with SARS-CoV-2. MISTRG6-hACE2 (+AAV): 2dpi n=2, 4dpi n=5, 7dpi n=2, 14dpi n=6 MISTRG6(−AAV): 2dpi n=4, 4dpi n=10 and 7, 14 dpi n=2, biologically independent mice representative of at least 2 independent experiments. Viral titers using standard Vero E6 cells do not have any detectable titers (previously reported^19^) in MISTRG6 mice without AAV. Some of the MISTRG6-hACE2 data presented here have been previously reported as part of the characterization of the model^19^. i. Frequencies of mNG+ cells within human macrophages (human CD68+) isolated from lungs of infected MISTRG6 mice transduced with AAV-hACE2 (AAV+) or not (AAV−). MISTRG6 mice with and without AAV-hACE2 were reconstituted with human progenitor cells from the same donor. AAV+ n=6, AAV− n=5 biologically independent mice examined over 3 independent experiments. j. Representative gating strategy for sorting mNG+ and mNG− human immune cells, mNG+ and mNG− mouse epithelial cells and mouse immune cells. Lung cells from SARS-CoV2-mNG infected MISTRG6-hACE2 mice were stained with antibodies against human CD45, mouse CD45, and mouse EPCAM. Sorted cells were used for viral quantification (Fig. 2) and characterization of the inflammasome pathway (Fig. 3).

**Extended figure 7.**
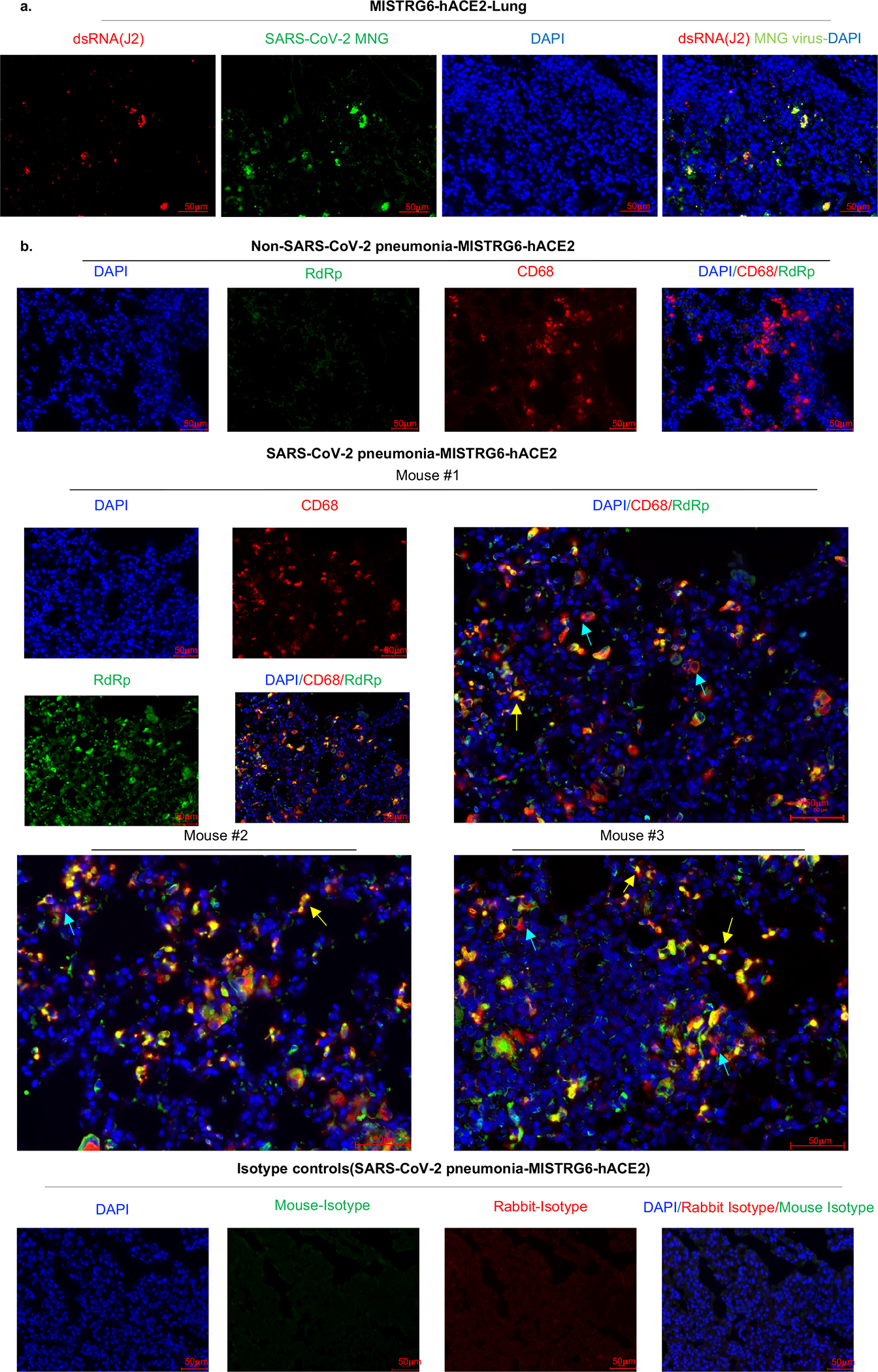
Viral replication products are detected in human lung macrophages of infected MISTRG6-hACE2 mice (matched to figure 2). a. Representative fluorescent microscopy images showing colocalization of double stranded RNA (clone rJ2) staining, mNG signal and DAPI staining in fixed lung tissue at 4dpi. Representative of n=4 biologically independent mice examined over 2 independent experiments. b. Representative fluorescent microscopy images of RNA dependent RNA polymerase (RdRp), anti-human CD68 and DAPI staining in fixed lung tissue from SARS-CoV-2 infected or control MISTRG6-hACE2 mice (Non-SARS-CoV-2 pneumonia). Representative of n=7 biologically independent SARS-CoV-2 infected mice examined over 3 independent experiments. Yellow arrows mark RdRp+ human macrophages. Blue arrows mark RdRp− human macrophages. Isotype controls (bottom panels) and non- COVID pneumonia lungs (bacterial infection, top panels) n=3 biologically independent mice are presented as controls. Pseudo-colors were assigned for visualization.

**Figure 2.**
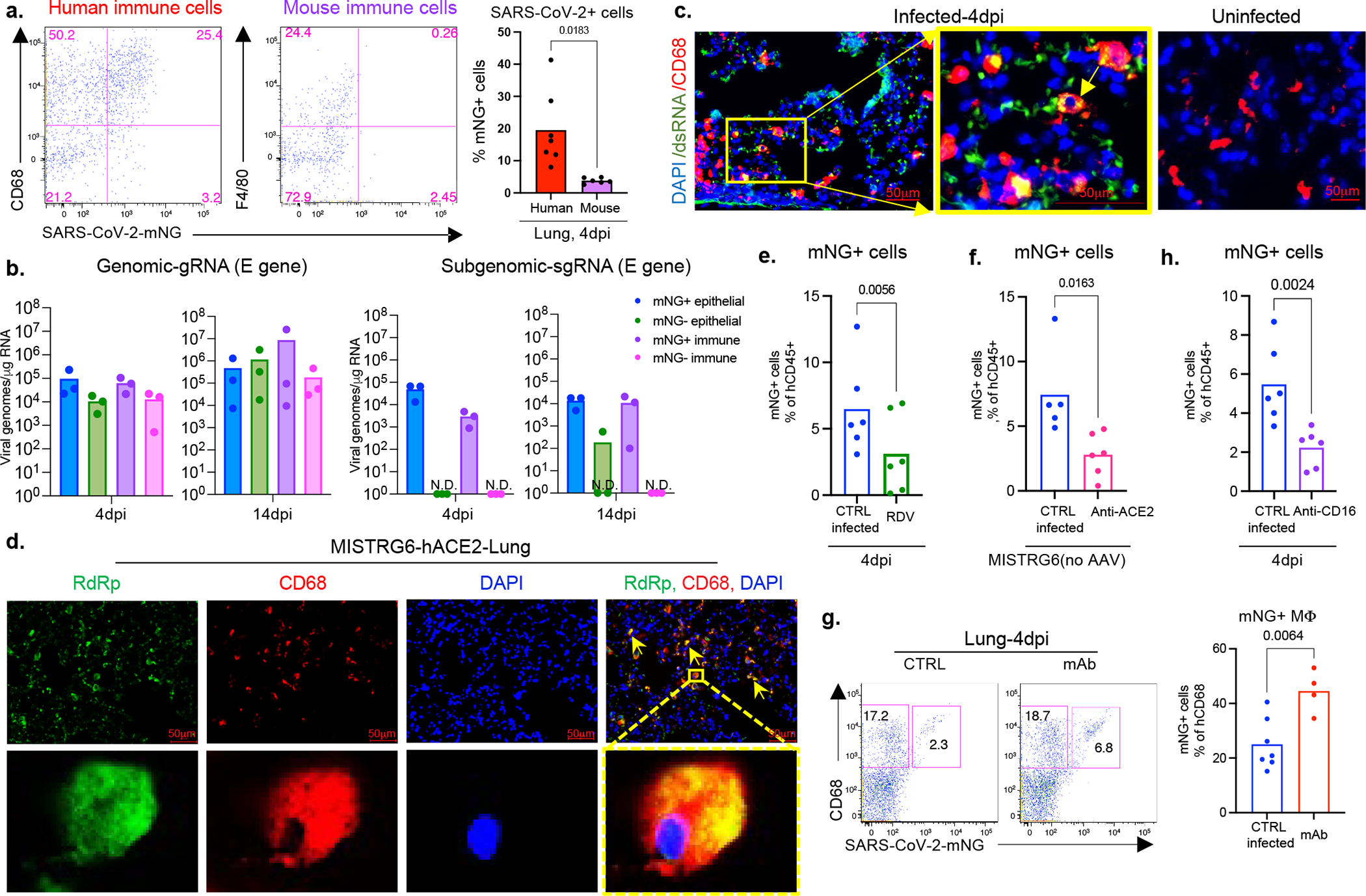
SARS-CoV-2 replicates in human macrophages. a. Representative flow cytometry plots and frequencies of mNG+ human (CD68+) or mouse (F4/80+) lung macrophages in SARS-CoV-2-mNG infected MISTRG6-hACE2 mice. Human n=7, mouse n=6 mice over at least 3 experiments. Unpaired, two-tailed t-test. b. Quantification of gRNA and sgRNA (E-gene)^40,69^ in sorted mNG+ or mNG− epithelial cells or human immune cells. N=3 mice over 2 experiments. Means with datapoints. c. Representative fluorescent microscopy images of dsRNA (rJ2), CD68 and DAPI staining in fixed lung tissues from SARS-CoV-2 infected MISTRG6-hACE2 mice. Representative of n=5 mice examined over 3 experiments. Yellow rectangle: higher magnification view of the selected area. Yellow arrow: colocalization of CD68 with dsRNA. Pseudo-colors were assigned. d. Representative fluorescent microscopy images of RdRp, CD68 and DAPI staining in fixed lung tissues from SARS-CoV-2 infected MISTRG6-hACE2 mice. Representative of n=5 mice examined over 3 experiments. Yellow arrows: colocalization of human CD68 with dsRNA. Yellow rectangle: higher magnification view of the selected area. Pseudo-colors were assigned. e. Frequencies of mNG+ human immune cells in Remdesivir treated (1–3dpi) or control MISTRG6-hACE2 mice infected with SARS-CoV-2-mNG. N=6 mice examined over 3 experiments. Means with datapoints. Paired t-test, two-tailed. f. Frequencies of mNG+ human immune cells upon ACE2 blockade (1–3dpi) in MISTRG6 (no AAV) mice infected with SARS-CoV-2-mNG. CTRL-infected n=5, anti-ACE2 treated n=6 mice examined over 2 experiments. Means with datapoints. Paired t-test, two-tailed. g. Representative flow cytometry plots and frequencies of mNG+ macrophages in infected MISTRG6-hACE2 mice treated with monoclonal antibodies (mAb)^19,45,64^ at 35hpi. CTRL-infected n=7, treated n=4 mice examined over 2 experiments. Means with datapoints. Unpaired, two-tailed t-test. h. Frequencies of mNG+ human immune cells in MISTRG6-hACE2 mice after CD16 blockade (Abcam, 2dpi). N=6 mice examined over 3 experiments. Means with datapoints shown. Paired t-test, two-tailed.

To determine the cellular source of persistent viral RNA and replication, we measured genomic (gRNA) and subgenomic viral RNA (sgRNA)^40^ in lung tissue or in sorted lung epithelial cells or human immune cells from infected MISTRG6-hACE2 mice (Extended Data Fig. 6a–d). Surprisingly, epithelial cells and human immune cells had similar levels of viral RNA (Extended Data Fig. 6d). Although gRNA was abundant, we could not discern sgRNA in either cell type. We tracked infected cells in MISTRG6-hACE2 mice using a reporter strain of virus, SARS-CoV-2-mNG^41^, which encodes the fluorescent protein mNG in infected cells. By this assay, most epithelial cells in bronchioalveolar lavage (BAL) but only few total lung epithelial cells were infected with SARS-CoV-2 (Extended Data Fig. 6e). Strikingly, human macrophages were strongly mNG positive throughout disease (Fig. 2a, Extended Data Fig. 6f,g). No mouse immune cells expressed mNG (Fig. 2a, Extended Data Fig. 6f). To address whether the SARS-CoV-2 viral RNA replicates in these cells or is acquired by phagocytosis, we measured the mNG signal in human macrophages from infected MISTRG6 mice untransduced with hACE2. In these mice, epithelial cells were not infected or infected poorly with SARS-CoV-2^19,42^ (Extended Data Fig. 6h). These mice had, however, similar levels of mNG+ human macrophages as AAV-hACE2 mice, suggesting viral uptake by macrophages is independent of infected epithelial cells (Extended Data Fig. 6i). To determine whether SARS-CoV-2 replicates in human macrophages, we quantified gRNA and sgRNA^40^ in mNG+ vs mNG− epithelial or human immune cells at 4dpi or 14dpi (Extended data Fig. 6j). Only mNG+, not mNG−, epithelial and immune cells had sgRNA (Fig. 2b). Second, we stained for dsRNA, diagnostic of viral replication (Fig. 2c). As expected, mNG and dsRNA were detected/colocalized in human macrophages (Fig. 2c, Extended data Fig. 7a). Third, we detected viral RdRp in human macrophages, which colocalized with a viral Spike protein supporting specificity (Fig. 2d, Extended data figure 7b, 8a–c). Viral RdRp and Spike were also present in human macrophages of human autopsy lungs with SARS-CoV-2 pneumonia (Extended Data Fig. 9). Thus, the mouse model observations reflected human disease. Remdesivir reduced the mNG signal and viral titers by the same amount in infected MISTRG6-hACE2 mice (Fig. 2e, Extended Data Fig. 10a). Thus, SARS CoV-2 appeared to replicate in human immune cells.

### SARS-CoV-2 infects via ACE2 and CD16

The ACE2 receptor utilized by SARS-CoV-2 to infect lung epithelium can be expressed in macrophages^43^. We measured ACE2 expression by flow cytometry and immunofluorescence staining in mouse epithelial cells and human lung macrophages (Extended Data Fig. 10b–f). Human lung macrophages from both MISTRG6 and MISTRG6-hACE2 mice, but only epithelial cells from MISTRG6-hACE2 mice expressed human ACE2 (Extended Data Fig. 10b–e). Interestingly, ACE2 expression was higher in both infected (mNG+) human macrophages and epithelial cells (Extended Data Fig. 10b–e). We treated SARS-CoV-2 infected MISTRG6 mice with a blocking antibody against human ACE2. In these mice only, SARS-CoV-2 infects epithelial cells poorly^19,42^ as the mice did not receive AAV-ACE2 and only human macrophages express human ACE2 (Extended Data Fig. 6h). ACE2 blockade significantly diminished infected human macrophages (Fig 2f), suggesting ACE2 can mediate viral entry in human lung macrophages.

Antibodies can also mediate viral uptake by macrophages (e.g., Dengue virus^44^). To test the role of antibody-mediated viral entry to macrophages, we treated infected mice with monoclonal antibodies (mAb)^45^ against SARS-CoV-2-Spike protein early (35hpi) when effects of endogenous antibodies are minimal or late (7dpi) (Extended Data Fig. 10g). Indeed, mAb treatments increased infected lung macrophages (Fig. 2g, Extended Data Fig. 10h). Immune cells express a wide range of surface Fcγ receptors (FcγRs) which interact with the Fc moiety of antibodies. These interactions lead to multiple protective or pathological effector functions^44,46^. COVID-19 severity correlates with high serum IgG levels and specific IgG-Fc structures and interactions^47–49^. One such Fc-interaction is mediated by CD16, expressed at high levels in mNG+ macrophages. We treated mice early (2dpi, low antibody levels) as proof of concept, or late (7dpi and 11dpi, high antibody levels) as a possible therapeutic with anti-CD16 antibody. Anti-viral antibody levels in lung tissue were sufficient to mediate viral uptake and positively correlated with mNG levels at 4dpi (Extended Data Fig. 10g, i). With dosing optimized, CD16 blockade which did not alter distribution of macrophages, however resulted in significantly fewer infected human macrophages at both timepoints (Fig. 2h and Extended Data Fig. 10j).

To elucidate whether viral replication products are the result of bona-fide infection, we cultured bone-marrow derived macrophages (BMDMss) with SARS-CoV-2 *in vitro*. Indeed, SARS-CoV-2 were taken up by BMDMs and replicated in these cells as measured by mNG signal (Extended data Fig. 10k) and high levels of sgRNA (Extended data Fig. 10l). This was true for multiple types of macrophages (Extended data Fig. 10m). As *in vivo*, *in vitro* macrophage infection enhanced by antibodies (convalescent plasma or mAbs) was reduced by CD16, ACE2, or RdRp blockade (Extended data Fig. 10n,o). SgRNA levels in these macrophages were also reduced by these treatments (Extended data Fig. 10l), further supporting a role for both ACE2 and CD16 in viral uptake and RdRp in viral replication. SARS-CoV-2 infection in human macrophages was not productive or produced very little as indicated by undetectable infectious virus, titered in culture, from sorted immune cells from infected mice at 4dpi and *in vitro* infected macrophages at 48hpi (Extended data Fig. 10p–r).

### Transcriptome of infected macrophages

**Figure 3.**
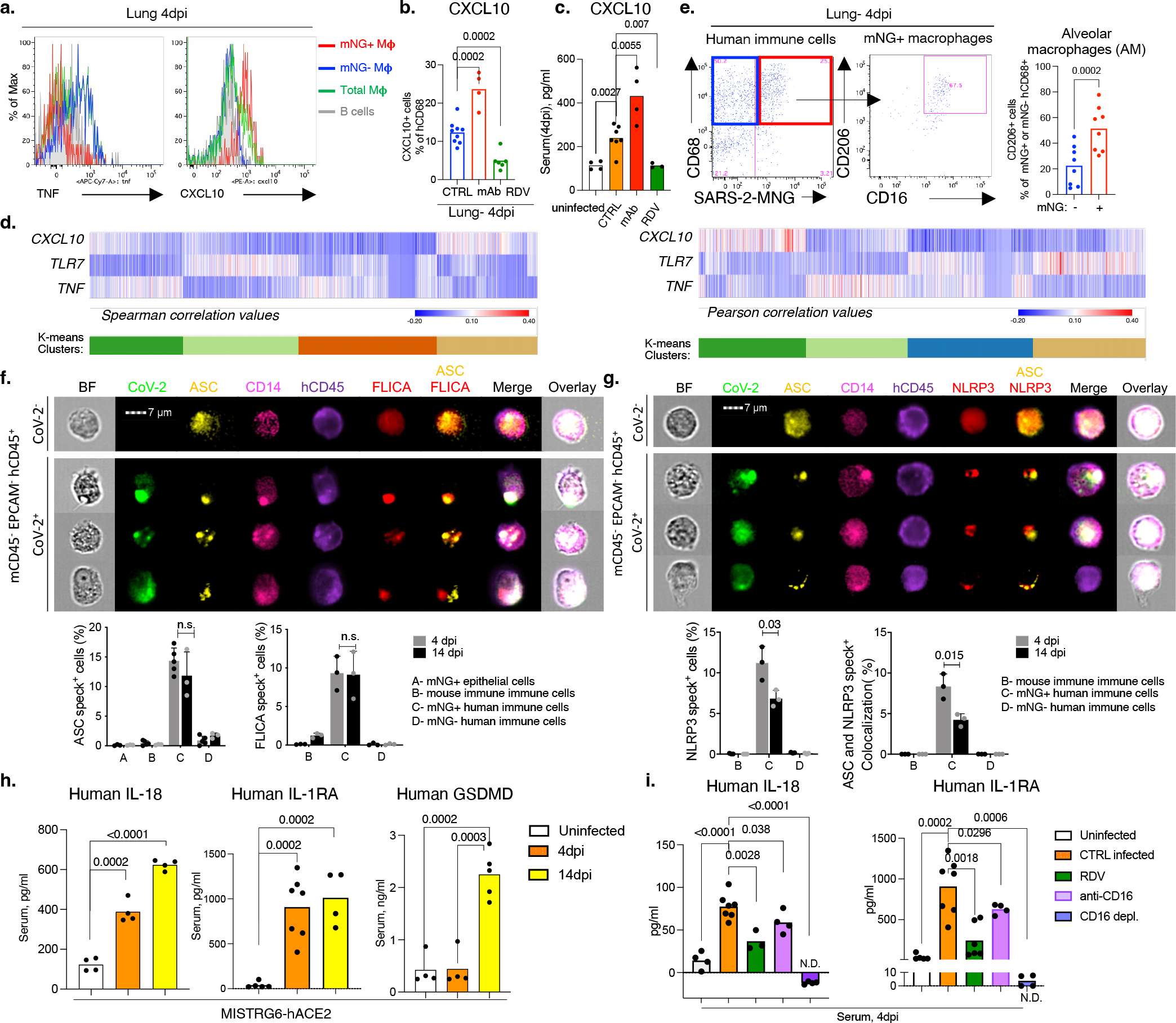
SARS-CoV-2 infection of human macrophages activates inflammasomes and pyroptosis. a. CXCL10+ or TNF+ human macrophages. Representative of n=6 mice over 3 experiments. b. CXCL10+ lung macrophage frequencies upon mAb or Remdesivir therapy. CTRL-infected n=9, mAb n=4, RDV n=6 mice over 2 experiments. Means with datapoints. Unpaired, two-tailed-t-test. c. Serum CXCL10 levels upon mAb or Remdesivir therapy. Means with datapoints. Uninfected, mAb n=4; CTRL-infected n=7, RDV n=3 mice examined over 2 experiments. Unpaired, two-tailed-t-test. d. Correlation (Pearson and Spearman) of each gene with *CXCL10*, *TNF* or *TLR7* in human lung monocytes and macrophages. K-means clustering. P-values: T-distribution with length(x)-2 degrees of freedom or algorithm AS 89 with exact = TRUE. Two-tailed. e. Representative plots and AM frequencies within mNG+ or mNG-macrophages. N=8 mice examined over 4 experiments. f. ASC speck visualization/quantification and colocalization with active caspase-1 (FLICA) in mNG+ or mNG-human immune cells from MISTRG6-hACE2 mouse lung. Cells sorted based on Extended data Fig. 6j. 1000-cells analyzed/per condition. ASC+specks: 4dpi n=3(A), 5(B-D); 14dpi n=3 mice, FLICA: n=3 mice examined over at least 2 experiments. Means with datapoints, SD. Unpaired, two-tailed-t-test. g. ASC speck visualization/quantification and colocalization with NLRP3 oligomerization in sorted mNG+ or mNG-human lung immune cells. 1000-cells analyzed/per condition. N=3 mice over 2 experiments. Means with datapoints, SD. Unpaired, two-tailed-t-test. h. Serum IL-18, IL-1RA and GSDMD levels. IL-18: n =4 mice examined over 2 experiments. IL-1RA: Uninfected n=5, 4dpi n=7, 14dpi n=4 mice examined over 3 experiments. GSDMD: uninfected, 4dpi n=4, 14dpi n=5 mice over 3 experiments. Means with datapoints. Unpaired, two-tailed t-test. P-value<0.0001=3.32×10^−7^. i. Serum IL-18 and IL-1RA levels in mice treated with CD16-blocking (Abcam) or depleting (ThermoFisher), antibodies or Remdesivir. IL-18: uninfected, CD16-blocking, CD16-depletion n=4, CTRL-infected n=7, RDV n=3; IL-1RA: uninfected n=5, CTRL-infected n=7, RDV n=6, CD16-blocking, CD16-depletion n=4 mice examined over at least 2 experiments. Means with datapoints. Unpaired, two-tailed t-test. P-values<0.0001:uninfected=3.28×10^−5^, CD16-depl=7.92×10^−7^.

We next determined the consequences of infection of human macrophages by SARS-CoV-2. Infected macrophages preferentially produced CXCL10, a chemokine which recruits many types of immune cells (Fig. 3a), but not TNF. Like mNG positivity itself, CXCL10 production by human macrophages was also enhanced by antibodies and inhibited by Remdesivir, also reflected in serum levels and *in vitro* (Fig. 3b,c, Extended data Fig. 11a–c). Thus, we used CXCL10 as a proxy for SARS-CoV-2-infected macrophages and determined a unique transcriptional signature enriched for genes encoded by tissue-resident macrophages, in particular AMs^50^ (*APOC1, MRC1, ALOX5AP, FABP5, INHBA*), chemokines of interstitial macrophages (*CCL18, CCL3, CCL7, CCL8, CCL20, CXCL8*), inflammatory cytokines (*IL1A, IL18, IL27*), complement genes (*C1QA, C1QB*) and ISGs (*ISG20, IFI27*) (Fig. 3d, Extended data Fig. 11d,e, 12a,b, Table S4). Further flow cytometric characterization of mNG+ cells also confirmed enrichment for CD16+ AMs, which produced more CXCL10 (Fig. 3e, Extended data Fig. 12c). In line with our findings, CD14^hi^CD16^hi^ cells and AMs enriched with viral RNA in autopsy lungs of COVID-19 patients^7,20^ also had distinct transcriptomes which were largely recapitulated in what we construe as CXCL10-associated genes (CXCL11, CCL18, CCL8, ISG15, CD83). Interestingly, this strong network of CXCL10 specific gene signature was no longer restricted to AMs later in infection as different macrophage subsets continuously differentiate, evident in high *IL7R* expression by developing lung macrophages^50^ (CXCL10+ and AM) at all timepoints (Fig. 1g, 3d, Extended data Fig. 12d).

### SARS-CoV-2 activates inflammasomes

Morphological analysis of sorted mNG+ cells revealed the appearance of membrane bubbles, a characteristic of pyroptosis and prompted us to investigate inflammasome activation as part of the inflammatory cascade initiated by infection. Inflammasomes are dynamic multiprotein complexes in which specific NOD-like receptors (NLRs) and adaptor molecules are assembled to activate caspases, the central effector proteins. We sorted mNG+ and mNG− human immune cells, mNG+ epithelial cells, and mouse immune cells (Extended data Fig. 6j) and assayed for sensors, adaptors, and effectors of the inflammasome pathway. First, focusing on adaptor molecule apoptosis-associated speck-like protein containing a CARD (ASC) as the common adaptor molecule with a pivotal role in inflammasome assembly and activation, we found that infected (mNG+) human cells exclusively showed significant inflammasome activation, quantified by ASC speck formation (Fig.3f, Extended Data Fig. 13a,b). ASC specks co-localized with both NLRP3 and active caspase-1 (visualized by fluorochrome-labeled inhibitor of caspases assay (FLICA)) (Fig 3f, g, Extended Data Fig. 13a–d). Inflammasome activation in infected human macrophages was sustained during disease (4–14dpi; Fig 3f,g, Extended Data Fig. 13c).

Once inflammasome complexes are formed, active caspase-1 cleaves and proteolytically activates the proinflammatory IL1-family cytokines, IL-1β and IL-18, typically elevated and characteristic of severe COVID-19 in patients. IL-18 levels in blood and lungs were significantly elevated in SARS-CoV-2 infected mice and correlated well with proportions of infected (mNG+) macrophages (Fig. 3h, Extended Data Fig. 13e). Although IL-1β levels in serum in vivo were not detectable, we measured IL-1RA. This specific receptor antagonist, induced by IL-1β, served as a proxy of IL-1β and it paralleled enhanced IL-18 levels and correlated with mNG+ cells (Fig 3h, Extended Data Fig. 13f).

Finally, we assayed for pyroptosis by detecting LDH and gasdermin D (GSDMD) in serum. GSDMD, a substrate of active caspase-1 and pore-forming executer of pyroptosis, and LDH, released by pyroptosis, were particularly enriched in serum of infected mice at late timepoints (14dpi-Fig.3h, Extended Data Fig. 13g), further supporting continuous inflammasome activation during infection. In addition, infected lung macrophages showed higher incorporation of a small fixable dye (Zombie Aqua) that enters dying cells with a compromised cell membrane, consistent with the pore-forming function of GSDMD and pyroptosis (Extended Data Fig. 13h).

All aspects of inflammasome activation were also recapitulated in vitro when BMDMs were infected in vitro with SARS-CoV-2. Active caspase-1 in infected BMDMs was dependent on viral replication was inhibited by Remdesivir (Extended Data Fig. 13i). High levels of inflammasome products IL-18, IL-1β, and IL-1RA (in response to IL-1β) and two measures of pyroptosis, GSDMD and LDH, were detected at high levels in supernatants of infected BMDMs were also inhibited by remdesivir (Extended data Fig. 13j–n). In vitro infected cells also had higher incorporation of Zombie-Aqua consistent with pyroptosis (Extended Data Fig. 13o).

### Infection impacts macrophage response

To determine the role of viral infection on the inflammatory macrophage response, we first blocked viral entry and replication in vivo and measured inflammatory cytokines and chemokines. Blocking viral entry (CD16 or ACE2 blockade) or inhibiting viral replication (Remdesivir) all reduced IL-18, IL-1RA, and CXCL10 levels, paralleling mNG levels (Fig 3i, Extended Data Fig. 14a–e). Depletion of CD16+ cells in vivo (Extended Data Fig. 14f,g) resulted in complete loss of IL-18 and IL-1RA in serum consistent with the concept that viral replication and inflammasome activation occurred mainly in myeloid cells (Fig 3i). On the other hand, mAbs which promoted viral infection of human macrophages (Fig. 2g, Extended data Fig. 10h) enhanced systemic IL-18, IL-1RA, and CXCL10 particularly in early disease (Extended Data Fig. 14h–k). Nonetheless, despite changes in levels of the inflammatory cytokines and chemokines, neither mAbs nor CD16 blockade impacted lung pathology potentially because of the conflicting role of these antibodies on viral titers vs. inflammation (Extended Data Fig. 14l, m). In line with the in vivo studies, IL-18, IL-1β, IL-1RA, and CXCL10 levels were also reduced in supernatants of in vitro infected BMDMs upon CD16, ACE2 or RdRp inhibition, again paralleling the reduced viral replication inferred from mNG levels (Extended Data Fig. 10o, 14n–q).

### Inflammasome inhibition in COVID-19

**Figure 4.**
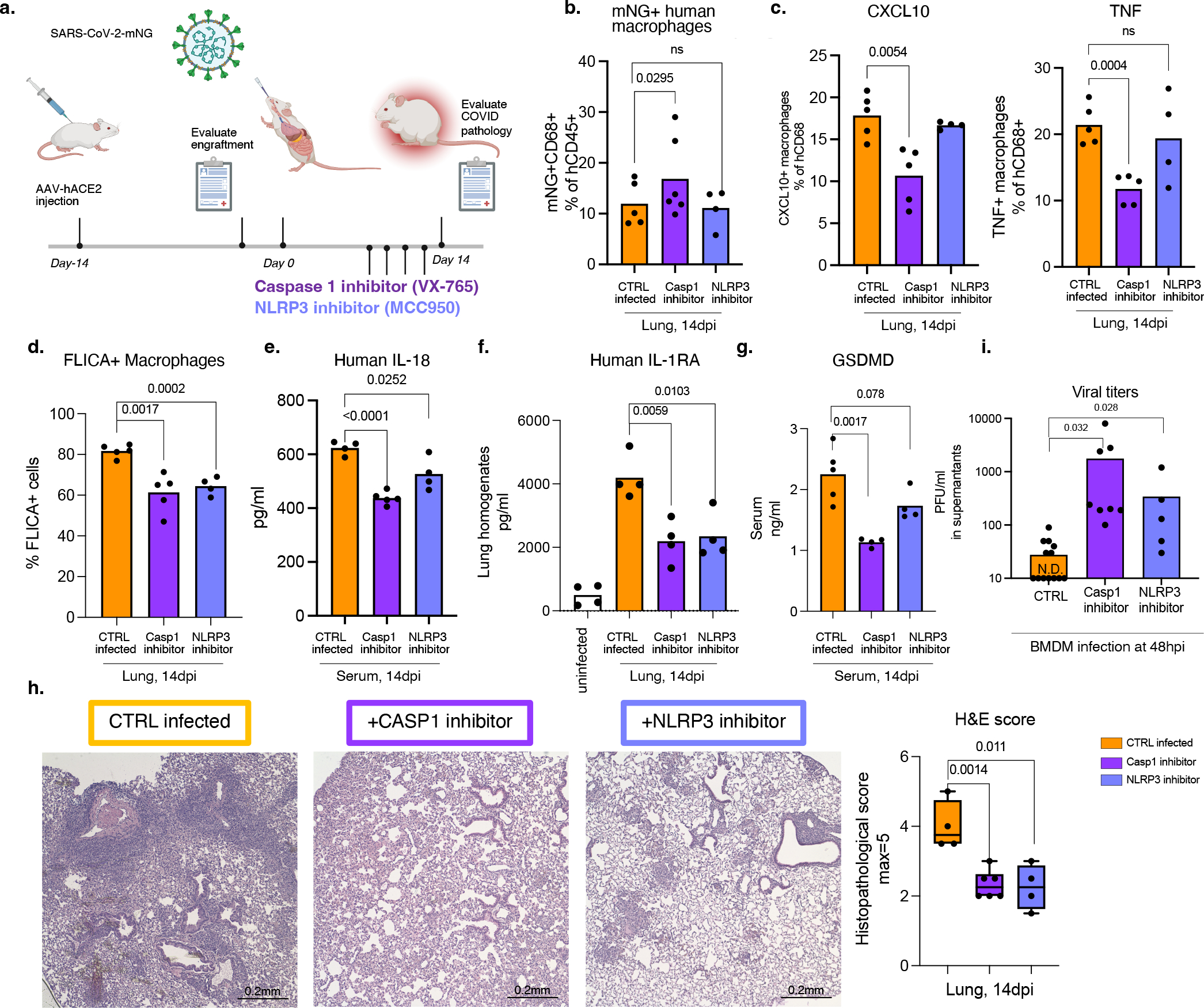
Inflammasome inhibition ameliorates inflammation and disease in infected MISTRG6-hACE2 mice. a. Schematic of inflammasome inhibition *in vivo*. SARS-CoV-2 infected MISTRG6-hACE2 mice treated with caspase-1 or NLRP3 inhibitors, 6–12dpi. b. Frequencies of mNG+ human immune cells upon inflammasome inhibition. CTRL-infected n=5, caspase-1 inhibitor n=6, NLRP3 inhibitor n=4 mice examined over at least 2 experiments. Means with datapoints. Paired, two-tailed t-test. c. Frequencies of CXCL10+ or TNF+ human lung macrophages upon inflammasome inhibition. CTRL-infected n=5, caspase-1 inhibitor n=5, NLRP3 inhibitor n=4 mice examined over at least 2 experiments. Means with datapoints. Unpaired, two-tailed t-test. d. Quantification of active caspase-1 in mNG+ human macrophages upon inflammasome inhibition. CTRL-infected n=5, Casp1-inhibitor n=5, NLRP3-inhibitor n=4 mice examined over at least 2 experiments. Means with datapoints. Unpaired, two-tailed t-test. e. Serum human IL-18 levels upon inflammasome inhibition. CTRL, NLRP3: n=4, Casp1 n=5 mice examined over 2 experiments. Means with datapoints. Unpaired, two-tailed t-test. P<0.0001=1.00114×10^−5^. f. Human IL-1RA levels in lung homogenates upon inflammasome inhibition. N=4 mice examined over 2 experiments. Means with datapoints. Unpaired, two-tailed t-test. g. Serum GSDMD levels upon inflammasome inhibition. CTRL-infected n=5, Casp1, NLRP3 inhibitors n=4 mice examined over 2 experiments. Means with datapoints. Unpaired, two-tailed t-test. h. Box and whisker plot of histopathological scores upon inflammasome inhibition. CTRL-infected n=4, Casp1-inhibitor n=6, NLRP3-inhibitor n=4 independent mice over at least 2 experiments. Whiskers: smallest (minimum) to the largest value (maximum). Box: 25^th^-75^th^ percentiles. Center line: median. Unpaired, two-tailed t-test. i. Viral titers from supernatants of BMDMs infected with SARS-CoV-2-mNG *in vitro* and treated with Casp1 or NLRP3 inhibitors. CTRL-infected: n=13, Casp1-inhibitor-: n=8, NLRP3 inhibitor: n=5 independent datapoints collected over 3 experiments. Means with datapoints. Unpaired two-tailed t-test.

Finally, to assess the causal role of NLRP3 and caspase-1 activation in inflammasome mediated inflammation and disease, we treated mice with caspase-1 and NLRP3 inhibitors (Fig.4a). As expected, the proportion of infected cells did not diminish (Fig. 4b), but the inflammatory profile of these cells and other lung macrophages was drastically attenuated (Fig. 4c). In inhibitor treated mice, mNG+ cells produced less CXCL10 which was also reflected in reduced serum levels (Fig. 4c, Extended Data Fig. 15a–c). Lung macrophages (mNG−) also produced less TNF (Fig. 4c, Extended Data Fig. 15a). Overall, inhibitor treated mice had lower levels of caspase-1 activation, IL-18, IL-1RA and GSDMD levels (Fig. 4d–g). The cumulative decrease in proinflammatory cytokines and chemokines upon inflammasome inhibition reversed the immune-pathological state of the lung, measured by scoring of lung histopathology (Fig. 4h). Inflammasome inhibition reduced immune cell infiltration and enhanced tissue recovery to homeostasis in lungs despite persistently high levels of mNG+ human immune cells in lungs.

Caspase-1 and NLRP3 inhibitors blocked inflammasomes *in vitro* but did not impact macrophage infection, measured as mNG+ macrophage frequency, and reduced the inflammatory response to infection (Extended Data Fig. 15d). All parameters of inflammasome activation: active caspase-1, IL-1β, IL-18, GSDMD and LDH were significantly reduced upon caspase-1 and NLRP3 inhibition *in vitro* (Extended Data Fig. 15e–i). Consistent with decreased pyroptosis, inflammasome blockade significantly reduced Zombie Aqua positive cells (Extended Data Fig. 15j). As seen *in vivo*, in*-vitro* infected BM macrophages produced less CXCL10 and IL-1RA (Extended Data Fig. 15k,l).

Finally, we tested whether inflammasome activation translated to any changes in infectious virus levels. We therefore first measured viral titers in lungs of caspase-1 inhibitor treated mice. Indeed, caspase-1 treated mice had higher viral load at 14dpi *in vivo* (Extended Data Fig. 15m). Given that, reduced inflammatory response could result in deficient viral clearance, we infected macrophages in vitro and treated them with caspase-1 or NLRP3 inhibitors to test the direct effect of inflammasome activation on infectious virus. Analysis of supernatants of these cultures showed that inhibitor treated cells produce substantially higher amounts of virus than the uninhibited controls (Fig. 4i, Extended Data Fig. 15n). Thus, the activation of inflammasomes in infected macrophages plays two protective functions- attenuates virus production and signals infection to the immune system by releasing inflammatory cues to recruit and activate more immune cells at the site of infection.

Overall, these findings suggest that infection of macrophages by SARS-CoV-2 activates inflammasomes and drives pyroptosis. Pyroptosis interrupts the viral replication cycle and prevents viral amplification; in parallel it releases immune cell activators and recruiters. Viral RNA/PAMPs and proinflammatory cytokines released from these cells likely shape the hyperinflammatory macrophage response sustained by infiltrating monocytes and MDMs and drive immunopathology.

## Discussion:

The MISTRG6 COVID-19 model faithfully reflects many of the chronic immunoinflammatory features of the human disease such as chronic viral RNA, IFN-response, and inflammatory state in macrophages^19^. Overall, our mechanistic study of this model defines a cascade of events, which initiates with infection of lung macrophages generating replicative intermediates and products including RdRp, dsRNA, sgRNA. SARS-CoV-2 replication activates an inflammatory program with activation of inflammasomes, production, and release of inflammatory cytokines and chemokines, and pyroptosis. We established all steps of inflammasome activation by visualizing ASC oligomerization, colocalization with active caspase-1 and NLRP3, maturation of inflammasome-mediated cytokines IL-1β and IL-18, and pyroptosis assayed by GSDMD and LDH release. Inhibitors of both caspase-1 and NLRP3 blocked the downstream aspects of inflammasome activation and the inflammatory cascade both *in vivo* and *in vitro*. More importantly, targeting inflammasome mediated hyperinflammation or combined targeting of viral replication and the downstream interferon response in the chronic phase of the disease prevented immunopathology associated with chronic SARS-CoV-2 infection *in vivo*.

Unlike epithelial cells, infected macrophages produce little virus. Strikingly however, inhibition of the inflammasome pathway led to a substantial increase in infectious virus produced by infected macrophages; though the degree these macrophages contribute, if at all, to high titers of virus production is unclear. Remarkably, inflammasome activation denies the virus the opportunity to replicate productively in these sentinel immune cells, and instead broadcasts inflammatory signals which inform the immune system of the infectious menace. While this is potentially beneficial, excessive inflammation that occurs through this mechanism coupled with the dysregulated interferon response may be the key factor which leads to the excessive inflammation that typifies chronic COVID-19^2,5,51–54^. Indeed, attenuation of the inflammasome *in vivo* blocks the inflammatory infiltrates in the lungs of infected mice *in vivo*. We speculate that, by contrast, an early interferon response, as may occur in the majority of patients who rapidly clear infection, and in the acute mouse models of infection where human immune cells that can be infected are not present, leads to viral elimination before this inflammatory chain reaction can occur.

Viral RNA and particles can be detected by a variety of innate immune sensors. Inflammasome sensor NLRP3 is both upregulated and activated by replicating SARS-CoV-2. NLRP3 inflammasome can directly sense viral replication/RNA or can rely on other viral RNA sensors like MDA5 or RIG-I^55–57^. Loss of IL-18/IL-1β production upon Remdesivir treatment in our studies strongly suggest viral replication is involved. Recent reports have also identified a possible role for NLRP3 driven inflammasome activation in SARS-CoV-2 infected myeloid cells in post-mortem tissue samples and PBMC^58^. Although many candidates have been proposed (lytic cell death upon infection, N protein^59^, ORF3A^60^), the exact mechanism of NLRP3 activation is still poorly understood^29^. Activation of other NLRs may also contribute to the process, as inhibition of caspase-1 was stronger than NLRP3 alone. Finally, there may be other mechanisms that enhance SARS-CoV-2 infection or the downstream inflammatory response in human macrophages that are unexplored here.

A role for inflammasome driven hyperinflammation in COVID-19 pathophysiology in patients is now recognized ^5–7,14–18^. Targeting inflammasome pathways in patients may provide alternative therapeutic options for resolving chronicity in COVID-19. However, the increased virus production seen upon inflammasome blockade could pose a significant risk to the benefit of wholesale inhibition of the pathway. The findings from our study and its implications provide alternative therapeutic avenues to be explored in the clinic and may guide novel therapeutic developments and prompt clinical trials to investigate combinatorial therapies that target viral RNA, inflammasome activation or its products and sustained IFN response.

## Data Availability

All data that support the findings of this study are available within the paper, its supplementary Information files, and source files. All 10x Genomics single cell RNA sequencing and bulk RNA sequencing data that support the findings of this study are deposited in the Gene Expression Omnibus (GEO) repository with accession codes GSE186794 and GSE199272.

## Materials and Methods

### Mice

MISTRG6 was generated by the R. Flavell laboratory by combining mice generated by this lab, the laboratory of Markus Manz and Regeneron Pharmaceuticals based on the *Rag2−/− IL2rg−/−*129xBalb/c background supplemented with genes for human M-CSF, IL-3, SIRPα, thrombopoietin, GM-CSF and IL-6 knocked into their respective mouse loci^61,62^. MISTRG6 mice are deposited in Jackson Laboratories and made available to academic, non-profit, and governmental institutions under a Yale-Regeneron material transfer agreement (already approved and agreed to by all parties). Instructions on obtaining the material transfer agreement for this mouse strain will be available along with strain information and upon request. CD1 strain of mice acquired from Charles River Laboratories were used for cross-fostering of MISTRG6 pups upon birth to stabilize healthy microbiota. All mice were maintained under specific pathogen free conditions in our animal facilities (either Biosafety Level 1, 2 or 3) under our Animal Studies Committee-approved protocol. Unconstituted MISTRG6 mice were maintained with cycling treatment with enrofloxacin in the drinking water (Baytril, 0.27 mg/ml). All animal experimentations were performed in compliance with Yale Institutional Animal Care and Use Committee protocols. For SARS-CoV-2–infected mice, all procedures were performed in a Biosafety Level 3 (BSL-3) facility with approval from the Yale Institutional Animal Care and Use Committee and Yale Environmental Health and Safety.

### Transplantation of human CD34+ hematopoietic progenitor cells into mice

Fetal liver samples were cut in small fragments, treated for 45 min at 37 °C with collagenase D (Roche, 200 μg/ml), and prepared into a cell suspension. Human CD34+ cells were purified by performing density gradient centrifugation (Lymphocyte Separation Medium, MP Biomedicals), followed by positive immunomagnetic selection with EasySep™ Human CD34 Positive Selection Kit (StemCell). For intrahepatic engraftment, newborn 1–3-day-old pups were injected with 20,000 fetal liver CD34+ cells in 20 μl of PBS into the liver with a 22-gauge needle (Hamilton Company). All use of human materials was approved by the Yale University Human Investigation Committee.

### AAV-hACE2 administration

AAV9 encoding hACE2^19,63^ was purchased from Vector Biolabs (AAV9-CMV-hACE2). Animals were anaesthetized using isoflurane. The rostral neck was shaved and disinfected. A 5-mm incision was made, and the trachea was visualized. Using a 32-G insulin syringe, a 50-μl injection dose of 10^11^ genomic copies per milliliter of AAV-CMV-hACE2 was injected into the trachea. The incision was closed with 4–0 Vicryl suture and/or 3M Vetbond tissue adhesive. Following administration of analgesic animals were placed in a heated cage until full recovery. Mice were then moved to BSL-3 facilities for acclimation.

### *In vivo* SARS-CoV-2 infection

SARS-CoV-2 isolate USA-WA1/2020 was obtained from BEI reagent repository. SARS-CoV-2 mNG was obtained from Dr. P.Y. Shi (UTMB)^41^. All infection experiments were performed in a BSL-3 facility, licensed by the State of Connecticut and Yale University. Mice were anesthetized using 20% vol/vol isoflurane diluted in propylene glycol. Using a pipette, 50 μl of SARS-CoV-2-WA1 or SARS-CoV-2-mNG (1–3×10^6^ PFU) was delivered intranasally.

### Therapeutics

SARS-CoV-2 infected MISTRG6-hACE2 were treated intraperitoneally daily with dexamethasone at 10mg/kg for 3 days starting at 7dpi. Mice were treated subcutaneously with Remdesivir at 25mg/kg dosing as has been previously described^22^ for 3 consecutive days starting at 7dpi (Fig. 1) or 1dpi (Fig. 2- for human macrophage infection studies mice were treated twice, daily). Mice were treated with anti-IFNAR2 antibody at 1.5mg/kg dosing on days 7 and 11 post infection. Weight changes post-infection were plotted as percent change compared with pre-infection weight.

Infected MISTRG6-hACE2 mice were treated with two different clones of anti-human CD16 antibodies. For CD16 blockade experiments, mice were treated with anti-CD16 (Abcam, clone SP175) antibody early and late. For early CD16 blockade studies mice were treated with anti-CD16 antibody at 2dpi with a single dose (20μg per mouse) and euthanized at 4dpi. For late CD16 blockade studies mice were treated with anti-CD16 antibody at 7dpi and 11dpi and euthanized at 14dpi. For depletion experiments mice were treated with anti-CD16 (ThermoFisher, clone 3G8) antibody with a daily dose of 20μg for 3 days starting 1dpi. Rabbit IgG, monoclonal [EPR25A] Isotype Control (ab172730) and Mouse IgG1 kappa Isotype Control (P3.6.2.8.1) were used.

Infected MISTRG6 (without AAV-hACE2) mice were treated with monoclonal antibody against human ACE2 (clone MM0073-11A31, Abcam-ab89111) for 3 days i.p. with a daily dose of 20μg starting at 1dpi. In these mice only, epithelial cells were not infected or infected poorly with SARS-CoV-2 with undetectable titers using standard plaque assays^19^ presumably due to differences between mouse and human ACE2 that limit viral entry and replication^42^. Mouse IgG2 Isotype was used as control.

Infected MISTRG6-hACE2 mice received a mixed cocktail of monoclonal antibodies clone 135 (m135) and clone 144 (m144) at 20mg/kg at 35hpi or 7dpi. Monoclonal recombinant antibodies (mAbs) used in this study were cloned from the convalescent patients (whose plasma was used for in vitro studies infecting BMDMs) and had high neutralizing activity against SARS-CoV-2 *in vitro* and *in vivo* in mouse adapted SARS-CoV-2 infection and ancestral stain of SARS-CoV-2/WA1^19,45,64^.

For NLRP3 inhibitor experiments, Infected MISTRG6-hACE2 mice were treated with MCC950 (R&D Systems) at a dose of 8 mg/kg intraperitoneally on days 6, 8, 10,12- post infection and euthanized on day 14^65–67^. For caspase-1 inhibitor experiments, infected MISTRG6-hACE2 mice treated with VX-765 (Invivogen) at a dose of 8 mg/kg on days 6, 8, 10,12 post infection and euthanized on day 14^67^. Control infected mice were treated with PBS.

### Viral titers

Mice were euthanized in 100% isoflurane. Approximately half of the right lung lobe was placed in a bead homogenizer tube with 1 ml of DMEM+2% FBS. After homogenization, 300 μl of this mixture was placed in 1mL Trizol (Invitrogen) for RNA extraction and analysis. Remaining volume of lung homogenates was cleared of debris by centrifugation (3,900 g for 10 min). Infectious titers of SARS-CoV-2 were determined by plaque assay in Vero E6 (standard) or Vero ACE2+TMPRSS2+ (sensitive) cells in DMEM 4% FBS, and 0.6% Avicel RC-581^68^. Plaques were resolved at 48 hours after infection by fixing in 10% formaldehyde for 1 hour followed by staining for 1 hour in 0.5% crystal violet in 20% ethanol. Plates were rinsed in water to visualize plaques. Multiple dilutions of lung homogenates were used to quantify Infectious titers (minimum number of plaques that can be quantified= 10 per ml of lung homogenate or ml of supernatant). Viral titers from supernatants of bone-marrow derived macrophage cultures were determined by plaque assay in Vero ACE2+TMPRSS2+ (sensitive) cells following the same protocols described for lung homogenates. VERO C1008 (Vero 76, clone E6, Vero E6) were obtained from ATCC. Vero ACE2+ TMPRSS2+ cells were obtained from B. Graham (NIAID). None of the cell lines were authenticated or tested for mycoplasma contamination.

### Viral RNA analysis

RNA was extracted with the RNeasy mini kit (Qiagen) per the manufacturer’s protocol. SARS-CoV-2 RNA levels were quantified using the Luna Universal Probe Onestep RT-qPCR kit (New England Biolabs) and US CDC real-time RT-PCR primer/probe sets for 2019-nCoV_N1. For each sample, 1 μg of RNA was used. Subgenomic viral RNA was quantified using primer and probe sets targeting E gene as has been previously described^40,69^. The primer-probe sequences were as follows: E_Sarbeco_F primer: ACAGGTACGTTAATAGTTAATAGCGT (400 nM per reaction). E_Sarbeco probe _P1: FAM-ACACTAGCCATCCTTACTGCGCTTCG-BBQ (200nM per reaction); E_Sarbeco_R primer ATATTGCAGCAGTACGCACACA (400 nM per reaction); E leader specific primer sgLead-F: CGATCTCTTGTAGATCTGTTCTC (400 nM per reaction).

### Histology and immunofluorescence

Yale pathology kindly provided assistance with embedding, sectioning of lung tissue. A pulmonary pathologist reviewed the slides blinded and identified immune cell infiltration and other related pathologies. Paraffin embedded lung tissue (fixed in 4% paraformaldehyde for no more than 24 hours) sections were deparaffinized in xylene and rehydrated. After antigen retrieval with 10 mM Sodium Citrate pH 6 and permeabilization with 0.1% Triton-X for 10 min the slides were blocked with 5% BSA in PBS with 0.05% Tween 20 for an hour. Then the samples were stained with primary antibodies against SARS-CoV-2-dsRNA; SARS-CoV2-RNA-dependent RNA Polymerase, SARS-CoV-2-Spike, human CD68, human ACE2 their isotype controls diluted in 1%BSA overnight at 2–8 °C. The next day, the samples were washed and incubated with fluorescent secondary antibodies. After washes, samples were treated with TrueBlack lipofuscin autofluorescence quencher for 30 seconds and mounted on DAPI mounting media (Sigma). Images were acquired using Keyence BZ-X800 Fluorescence Microscope or Nikon ECLIPSE Ti Series Confocal Microscope. Pseudo-colors were assigned for visualization.

### Isolation of cells and flow cytometry

All mice were analyzed at approximately 9–14 weeks of age. Single cell suspensions were prepared from blood, spleen, bronchioalveolar lavage (BAL) and lung. Mice were euthanized with 100% isoflurane. BAL was performed using standard methods with a 22G Catheter (BD). Blood was collected either retro-orbitally or via cardiac puncture following euthanasia. BAL was performed using standard methods with a 22G Catheter (BD)^70^. Lungs were harvested, minced, and incubated in a digestion cocktail containing 1 mg/ml collagenase D (Sigma) and 30 μg/ml DNase I (Sigma-Aldrich) in RPMI at 37°C for 20 min with gentle shaking. Tissue was then filtered through a 70 or 100-μm filter. Cells were treated with ammonium- chloride-potassium buffer and resuspended in PBS with 1% FBS. Mononuclear cells were incubated at 4°C with human (BD) and mouse (BioxCell, BE0307) Fc block for 10 min. After washing, primary antibody staining was performed at 4°C for 20 min. After washing with PBS, cells were fixed using 4% paraformaldehyde. For intracellular staining, cells were washed with BD permeabilization buffer and stained in the same buffer for 45 min at room temperature. Samples were analyzed on an LSRII flow cytometer (BD Biosciences). Data were analyzed using FlowJo software.

For cell sorting experiments, single cell suspensions from digested lungs were stained with antibodies against human CD45, mouse CD45, mouse EPCAM and sorted using BD FACS Aria II which is contained in a Baker BioProtect IV Biological Safety Cabinet. Cell viability was assessed with DAPI when applicable.

For imaging flow cytometry, cells from SARS-CoV-2 infected humanized mice were sorted based on: human immune cells (hCD45+); mouse immune cells (mCD45+) or epithelial mouse cells (EPCAM+). A- mNG+ epithelial cells (SARS-CoV-2-mNG+ mCD45(PE)− EPCAM(APC)+ hCD45(PB)−; B-total mouse immune cells (mCD45(PE)+ EPCAM(APC)− hCD45(PB−); C- mNG+ human immune cells (SARS-CoV-2-mNG+ mCD45(PE)− EPCAM(APC)− hCD45(PB)+); D- mNG− human immune cells (SARS-CoV-2-mNG− mCD45(PE)-EPCAM(APC)− hCD45(PB)+). These sorted cells (epithelial or immune cells) were fixed in 4% paraformaldehyde (PFA) for at least 30 minutes. Fixed sorted cells (epithelial or immune cells) were permeabilized, stained with unconjugated primary antibodies for ASC (1:200, rabbit), NLRP3 (1:200, goat), then stained with secondary antibodies (donkey anti-rabbit or goat conjugated with AlexaFluor 546 or 647, at 1:1000). Cells data were acquired using an ImageStream X MKII (Amnis) with 63X magnification and analyzed using Ideas software (Amnis). ASC, NLRP3 specks were gated and quantified based on fluorophore intensity/max pixels. For FLICA-Caspase1 colocalization, macrophages were pretreated with FLICA prior to sorting.

### *In vitro* infection with SARS-CoV-2

Using aseptic techniques under sterile conditions, bone marrow cells were isolated from femurs of immune-reconstituted MISTRG6 mice. For differentiation into human macrophages, bone marrow cells were incubated in media supplemented with 10% FBS, 1% penicillin/streptomycin and recombinant human M-CSF (50ng/ml), GM-CSF (50ng/ml) and IL-4 (20ng/ml) at 1×10^6^ per ml concentration for 6 days in 5% CO2 incubator at 37°C. Media supplemented with 10% FBS was replenished with new media every 3–4 days. Prior to infection, cells were monitored for granularity, elongated morphology, and stronger adherence to the plate. Purity was confirmed by flow cytometry. Human macrophages were then cultured with SARS-CoV-2-mNG in presence or absence of COVID patient plasma, healthy plasma, monoclonal antibodies (mix of clones 135 and 144, described as therapeutics), Remdesivir, anti-CD16 antibody, anti-ACE2 antibody, control isotype antibody, caspase-1 inhibitor (VX-765^71^) or NLRP3 inhibitor (MCC950).

*Ex vivo* lung macrophage cultures: To enrich for human macrophages and monocytes, lung cells from uninfected MISTRG6 mice were sorted based on CD11B and human CD45 expression. These cells were then incubated with GM-CSF and IL-4 for 48 hours to mature macrophages. Non-adherent cells were aspirated prior to culturing with SARS-CoV-2.

Bone-marrow derived macrophages (BMDMs) *in vitro* or lung macrophages *ex vivo* were cultured with a viral inoculum at 10^4 PFU of SARS-CoV-2-mNG (~ MOI=0.1). These macrophage cultures were then incubated at 37 C, 5% CO2 for 24, 48 and 72 hours at which time cells were harvested. Cells were dissociated from culture plate with 10 mM EDTA or Accutase (ThermoFisher) cell dissociation reagent (10–20 minutes). For studies pertaining to the mechanism of viral entry, viral replication and inflammasome activation, infected macrophages were treated with Remdesivir (10uM), anti-CD16 (Abcam clone, 10μg/ml) and anti-ACE2 (10 μg/ml), caspase-1 inhibitor (VX765, 20μM) and NLRP3 inhibitor (MCC950, 20μg/ml) in culture. Cells were stained when applicable and fixed for 30 min with 4% PFA. Convalescent plasma samples from the top 30 neutralizers in a cohort of 148 individuals were pooled to create a mixture with an NT50 titer of 1597 against HIV-1 pseudotyped with SARS-CoV-2 S protein^45^. We used this pooled serum at a concentration of 5μl-plasma/ml for *in vitro* experiments and refer to it as COVID patient plasma. Healthy plasma was collected from healthy volunteers and pooled prior to COVID-19 pandemic and used at a concentration of 5μl-plasma/ml. Monoclonal antibodies (a mix of clones 135 and 144) were used at 4 μg per ml concentration.

### Zombie Aqua and Annexin V staining

Single cell suspension from in vitro cultures or enzymatically dissociated lungs were washed and stained for viability with Zombie Aqua ((Biolegend- 423101) in PBS (1:400) for 15 min at 4°C. Without washing the cells, cell surface antibody cocktail was added, and cells were incubated for another 15 minutes. Cells were then washed with PBS twice and resuspended in Annexin V binding buffer. Cells were stained with Annexin V PE (1:400) in binding buffer for 15 min at 4°C. Cells were then washed with Annexin V buffer and fixed in 4% PFA.

### FLICA assay

Single cell suspension from in vitro cultures or enzymatically dissociated lungs were resuspended in RPMI 10% FBS with FLICA substrate (BioRad-FLICA 660 caspase-1 kit- ICT9122) and cultured for 1h (for microscopy) or 30 min (for flow cytometry) at 37°C. Cells were then washed twice with PBS and stained with Zombie Aqua and Annexin V as described. Cells were then fixed with 1x Fixative (provided in BioRad-FLICA caspase-1 kit) for at least 1 hour not exceeding 16 hours. Cells were kept at 4°C until further staining and analysis. FLICA 660 caspase-1 kit uses a target sequence (YVAD) sandwiched between a far-red fluorescent 660 dye (excitation max 660nm, emission max 685nm).

### LDH measurement

LDH levels were measured from freshly collected supernatant of infected cells (BMDMs) or freshly collected serum using CyQUANT LDH Cytotoxicity Assay (ThermoFisher- C20300) following manufacturer’s instructions under BSL3 conditions.

### Human Samples

For this study we have acquired six control uninfected, and two SARS-CoV-2 infected deidentified lung (4 different cuts) specimens as paraffin embedded tissues from autopsies of individuals admitted to Yale New Haven Hospital. Lungs were fixed in 10% Formalin (see Table S5 for details of patient specimens).

### Cytokine, chemokine, and IgG quantification

Human IL-18 (Sigma or RND), human CXCL10 (RND), human IL-1RA(Abcam), Human Gasdermin D (MyBioSource) were quantified from supernatants of BMDMs infected (or not) with SARS-CoV-2-mNG or from serum or lung homogenates of SARS-CoV-2-mNG infected (or not) MISTRG6 or MISTRG6-hACE2 mice following manufacturer’s instructions. Human IL-1β was quantified from supernatants of BMDMs infected with SARS-CoV-2-mNG using cytometric bead array for human IL-1B (BD) following manufacturer’s instructions. Human anti-Spike-RBD IgG (Biolegend) was quantified from sera and lung homogenates of infected or uninfected MISTRG6-hACE2 mice.

### Antibodies

#### Flow cytometry:

All antibodies used in flow cytometry were obtained from Biolegend, unless otherwise specified. Antibodies against the following antigens were used for characterization or isolation of cells by flow cytometry:

#### Mouse antigens:

CD45(Clone: 30-F11, Cat# 103130), CD45(Clone: 30-F11, Cat# 103108), CD45(Clone, 30-F11, Cat# 103147), CD326(Clone: G8.8, Cat# 118218), F4/80 (Clone: BM8, Cat# 123117).

#### Human antigens:

CD45(Clone: Hl30, Cat# 304044), CD45(Clone: Hl30, Cat# 304029), CD3(Clone: UCHT1, Cat# 300408), CD14(Clone: HCD14, Cat# 325620),CD16(Clone: 3G8, Cat# 302030), CD16(Clone: 3G8, Cat# 302006), CD19(Clone: HIB19, Cat# 302218), CD19(Clone: HIB19, Cat# 302226), CD33(Clone: WM53, Cat# 983902), CD20(Clone: 2H7, Cat# 302313), CD20(Clone: 2H7, Cat# 302322), CD206(Clone: 15–2, Cat# 321106), CD206(Clone: 15–2, Cat# 321109), CD86(Clone: BU63, Cat# 374210), CD123(Clone: 6H6, Cat# 306006), CD11B(Clone: M1/70, Cat# 101242), CD11C(Clone: 3.9, Cat# 301608), HLA-DR(Clone: LN3, Cat# 327014), HLA-DR(Clone: LN3, Cat# 327020), HLA-DR(Clone: LN3, Cat# 327005), CD183(Clone: G025H7, Cat# 353720), CD335-NKp46(Clone: 9E2, Cat# 331916), CD4(Clone: OKT4, Cat# 317440), CD8(Clone: SK1, Cat# 344718), CD8(Clone: SK1, Cat# 344748), CD68(Clone: Yl/82A, Cat# 333828).

#### Immunofluorescence:

Anti-dsRNA antibody (Clone: rJ2,) was purchased from Sigma (Cat# MABE1134) or Antibodies online (Cat# Ab01299–23.0). Polyclonal SARS-CoV-2 RNA-dependent RNA Polymerase antibody was purchased from CellSignaling (Cat# 67988S). Monoclonal SARS-CoV-2 RNA-dependent RNA Polymerase antibody was purchased from Kerafest (Cat# ESG004). Anti-Spike (Spike 1) antibody (clone: 1A9, Cat# GTX632604) was obtained from GeneTex. Anti-Spike (Spike 2) antibody (clone: T01Khu, Cat# 703958) was obtained from ThermoFisher.

#### Image Flow Cytometry:

Mouse anti-human PE-Cy7 CD16(Clone 3G8) was purchased from Biolegend (Cat# 302016). Rabbit anti-human ASC(Polyclonal) was purchased from Santa Cruz (Cat# sc-22514-R). Goat anti-human NLRP3(Polyclonal) was purchased from Abcam(Cat# ab4207). Donkey anti-Rabbit IgG (H+L) Highly Cross-Adsorbed Secondary Antibody(Polyclonal) was purchased from ThermoFisher (Cat# A-31573). Donkey anti-Rabbit IgG (H+L) Cross-Adsorbed Secondary Antibody (Polyclonal) was purchased from ThermoFisher (Cat# A-10040). Donkey anti-Goat IgG (H+L) Cross-Adsorbed Secondary Antibody(Polyclonal) was purchased from ThermoFisher(Cat# A-21447).

#### Therapeutic antibodies:

Monoclonal antibody against human CD16 used in blocking experiments were purchased from Abcam (SP175). Monoclonal antibody against human ACE2 was purchased from Abcam. Anti-CD16 antibody used in depletion experiments was purchased from ThermoFisher (3G8). Monoclonal antibodies (clones 135 and 144) were acquired from M. Nussenzweig as has been previously described^45^. Anti-IFNAR2 antibody was purchased from PBL Assay science (Cat# 21385–1).

### Gene expression

RNA was extracted with the RNeasy mini kit (Qiagen) per the manufacturer’s protocol High-Capacity cDNA Reverse Transcription Kit was used to make cDNA. Quantitative reverse transcription PCR (qRT-PCR) was performed using an SYBR FAST universal qPCR kit (KAPA Biosystems). Predesigned KiCqStart primers for *DDX58, IL6, IFITM3, IRF7, IFIH1, IFNA6, IFNG* and *HPRT1* were purchased from Sigma.

### Bulk whole tissue RNA-sequencing

RNA isolated from homogenized lung tissue, also used for viral RNA analysis, was prepared for whole tissue transcriptome analysis using low input (14dpi) or conventional (28dpi) bulk RNA sequencing. Libraries were made with the help of the Yale Center for Genomic Analysis. Briefly, libraries were prepared with an Illumina rRNA depletion kit and sequenced on a NovaSeq. Raw sequencing reads were aligned to the human-mouse combined genome with STAR^72^, annotated and counted with HTSeq^73^, normalized using DESeq2^74^ and graphed using the Broad Institute Morpheus web tool. Heatmaps visualize normalized counts of duplicates as min-max transformed values, calculated by subtracting row mean and diving by SD for each gene. Rows (genes) were clustered by hierarchical clustering (one-minus Pearson) or K-means clustering as indicated in the figure legends. Differential expression analysis was also performed with DESeq2. For IFN-stimulated gene identification, http://www.interferome.org was used with parameters -*In Vivo*, -Mus musculus or Homo sapiens -fold change up 2 and down 2.

### Single Cell RNA Sequencing 10X Genomics

Sorted human lung immune cells (hCD45+ in uninfected, 14dpi and 28dpi) were stained with TotalSeq (TotalSeq™-B0251 anti-human Hashtag 1 Antibody: GTCAACTCTTTAGCG; TotalSeq™-B0252 anti-human Hashtag 2 Antibody: TGATGGCCTATTGGG) antibodies (Biolegend) prior to processing for droplet based scRNA-seq. 10X Chromium GEM technology. Single cell transcriptomes and associated protocols of 4dpi lungs (total lung cells as opposed to sorted human immune cells analyzed) were previously described^19^. Duplicates from each condition/time point were pooled in equal numbers to ensure 10000 cells were encapsulated into droplets using 10X Chromium GEM technology. Libraries were prepared in house using Chromium Next GEM Single Cell 3ʹ Reagent Kits v3.1 (10X Genomics). scRNA-seq libraries were sequenced using Nova-Seq. Raw sequencing reads were processed with Cell Ranger 3.1.0 using a human-mouse combined reference to generate a gene-cell count matrix. To distinguish human and mouse cells, we counted the number of human genes (nHuman) and mouse genes (nMouse) with nonzero expression in each cell, and selected cells with nHuman > 20 * nMouse as human cells. The count matrix of human cells and human genes was used in the downstream analysis with Seurat 3.2^75^. Specifically, this matrix was filtered to remove low quality cells, retaining cells with > 200 and < 5,000 detected genes and < 20% mitochondrial transcripts. We then log normalized each entry of the matrix by computing log (CPM/100 + 1), where CPM stands for counts per million. To visualize the cell subpopulations in two dimensions, we applied principal component analysis followed by t-SNE, a nonlinear dimensionality reduction method, to the log-transformed data. Graph-based clustering was then used to generate clusters that were overlaid on the t-SNE coordinates to investigate cell subpopulations. Marker genes for each cluster of cells were identified using the Wilcoxon test (two-tailed) with Seurat. For the adjusted P values the Bonferroni correction was used. In this analysis, uninfected: 438 cells, 4dpi: 336 cells, 14dpi: 793 cells, 28dpi: 1368 cells were included.

To identify differentially abundant (DA) subpopulations not restricted to clusters, we applied DA-seq^76^, a targeted, multiscale approach that quantifies a local DA measure for each cell for comprehensive and accurate comparisons of transcriptomic distributions of cells. DA measure defined by DA-seq. shows how much a cell’s neighborhood is enriched by the cells from either uninfected or infected lungs. DA-seq analysis of our data revealed that T cells, monocytes and macrophages were responsible for most of the chronic infection driven changes. Red coloring signify enrichment at 28dpi lungs and blue coloring mark enrichment in uninfected lungs

To combine cells from different DPIs (uninfected, 4dpi, 14dpi, 28dpi), we applied the integration method^75^ in Seurat to remove batch effects. We then performed principal component analysis and retained top 30 PCs as the input to tSNE, a nonlinear dimensionality reduction method, to embed the data onto 2-dimensional space for visualization. Graph-based clustering with a resolution of 0.8 was then used to generate clusters that were overlaid on the t-SNE coordinates to investigate cell subpopulations. Marker genes for each cluster of cells were identified using the Wilcoxon test (two-tailed) with Seurat (For the adjusted P values the Bonferroni correction was used). After cell type identification, we separated out macrophage populations from all DPIs, and applied the same procedures as described above to re-preprocess and visualize the data. Clusters were redefined based on a resolution of 0.3.

### Statistics and Reproducibility

Unpaired or paired t-test (always two-tailed) was used to determine statical significance for changes in immune cell frequencies and numbers while comparing infected mice to uninfected control mice or treated mice to untreated mice. To determine whether the viral RNA quantification is statistically significant across treatment groups or timepoints, Mann-Whitney, two-tailed test was used. Unpaired t-test (two-tailed) or ratio paired t-test (two-tailed) was used to determine whether the viral titer quantification of the untreated condition is significantly different from the treated groups. For Pearson’s test, significance was deemed using t-test. The test statistic is based on Pearson’s product-moment correlation coefficient cor(x, y) and follows a t distribution with length(x)-2 degrees of freedom. For Spearman’s test, p-values are computed using algorithm AS 89 with exact = TRUE. All micrographs presented in the study were representative of at least 3 animals or specimens. Each experiment was repeated independently at least two times. All attempts yielded similar results. In *in vivo* studies, each dot represents a biologically independent mouse.

## Extended Data

**Extended figure 8.**
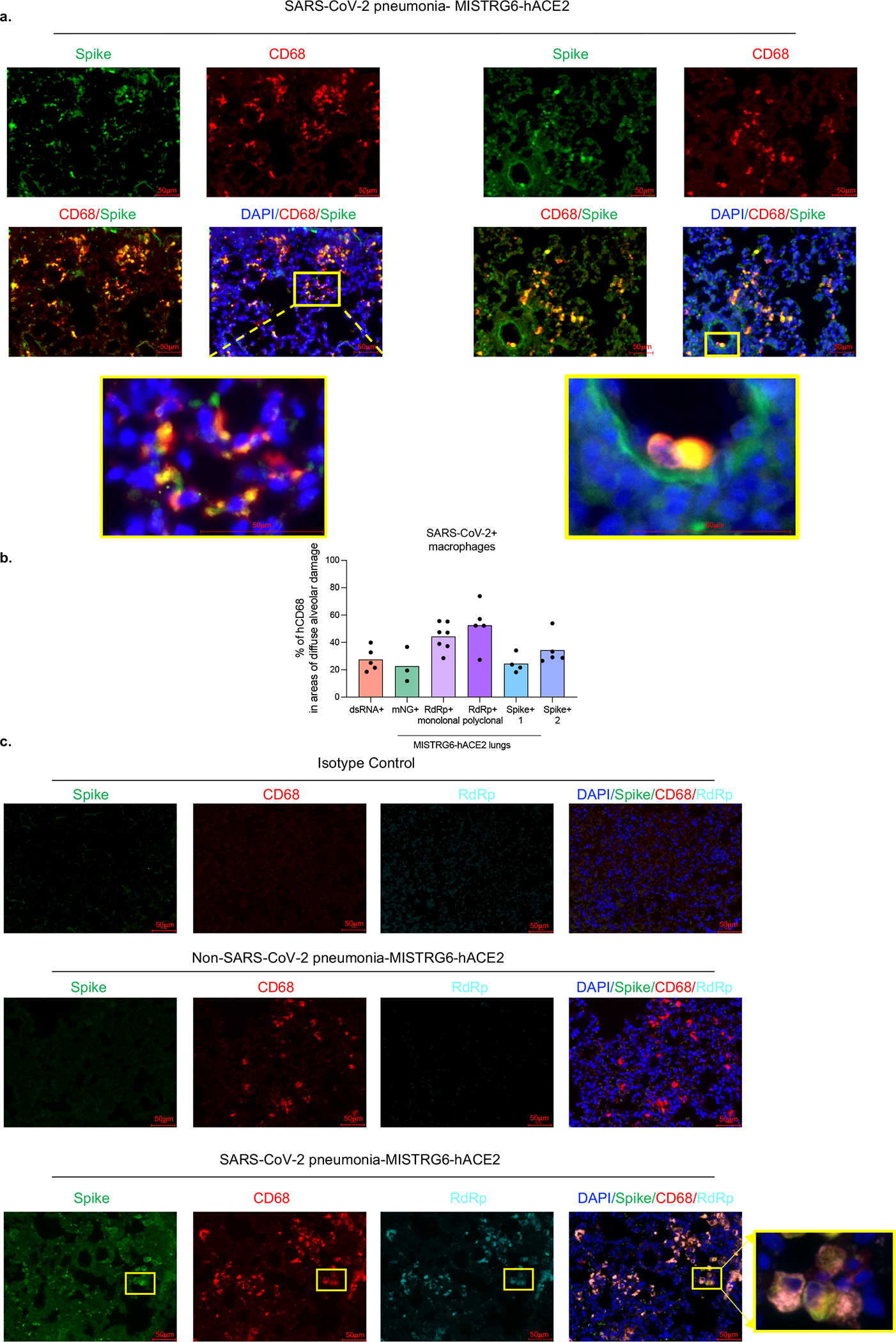
Viral RNA dependent RNA polymerase (RdRp) and Spike in human lung macrophages of MISTRG6-hACE2 mice infected with SARS-CoV-2 (matched to figure 2). a. Representative fluorescent microscopy images of Spike (S), human CD68, and DAPI staining in fixed lungs of SARS-CoV-2- infected MISTRG6-hACE2 mice. Yellow rectangle provides a higher magnification view of the selected area. Pseudo-colors were assigned for visualization. Representative of n=5 biologically independent mice examined over 3 independent experiments. b. Quantification of viral replication products or machinery in human lung macrophages from SARS-CoV-2 infected MISTRG6-hACE2 mice measured by immunofluorescent staining. Quantification was performed based on representative high-power images (40×) in areas showing diffuse alveolar damage. Frequencies of dsRNA, mNG, RdRp, and Spike positive human macrophages out of hCD68+DAPI+ cells are plotted. dsRNA: 20, 81, 133, 135, 52 human macrophages were counted. mNG: 30, 103, 110 human macrophages were counted. RdRp monoclonal: 187, 59, 85, 106, 142, 63, 59 human macrophages were counted. RdRp polyclonal: 134, 21, 22, 218, 44 human macrophages were counted. Spike (antibody 1): 21, 22, 218, 44 total human macrophages were counted. Spike (antibody 2): 63, 83, 163, 101, 57 human macrophages were counted. N=5 (dsRNA+), N=3 (mNG), N=7 (RdRp+ monoclonal), N=5 (RdRp+ polyclonal), N=4 (Spike-1), N=5 (Spike-2) biologically independent mice representative of at least 2 independent experiment. Means with all datapoints are shown. See supplementary methods for details of antibodies used. c. Representative fluorescent microscopy images and quantification of colocalization of Spike (S), RNA dependent RNA polymerase (RdRp), human CD68, and DAPI staining in fixed lungs of SARS-CoV-2-infected MISTRG6-hACE2 mice. Top panel: isotype control staining of SARS-CoV-2- infected lungs. Middle panel: control lungs with Non-SARS-CoV-2, bacterial pneumonia. Bottom panels: SARS-CoV-2-infected MISTRG6-hACE2 mice. Yellow rectangle provides a higher magnification view of the selected area. Pseudo-colors are assigned for visualization. Representative of n=5 biologically independent mice over 3 independent experiments.

**Extended data figure 9.**
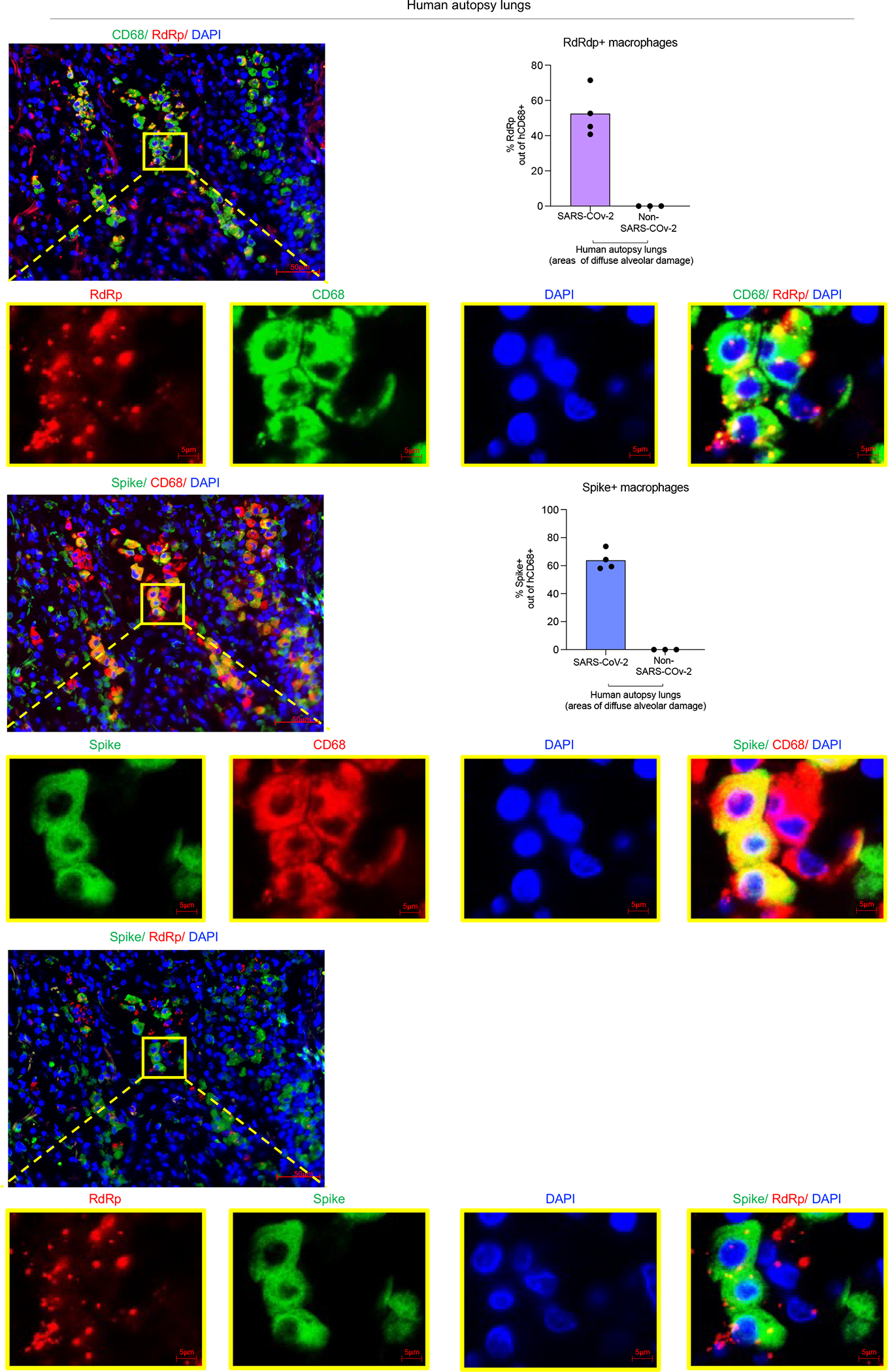
Viral RNA dependent RNA polymerase (RdRp) and Spike in human macrophages of human autopsy lungs with SARS-CoV-2 pneumonia (matched to figure 2). Representative fluorescent microscopy images and quantification of colocalization of Spike (S), RNA dependent RNA polymerase (RdRp), human CD68 and DAPI staining in fixed human autopsy lungs with SARS-CoV-2 pneumonia or non-SARS-CoV-2 pneumonia. Quantification was performed based on representative high-power images (40×) in areas showing diffuse alveolar damage. Top panels: Representative of RdRp staining with human CD68; middle panels: Representative of Spike staining with CD68; bottom panels: RdRp and Spike staining in SARS-CoV-2- infected autopsy lungs. Yellow rectangle provides a higher magnification view of the selected area. Pseudo-colors are assigned for visualization. SARS-CoV-2 pneumonia n=4, non-SARS-CoV-2 pneumonia n=3 biologically independent specimens.

**Extended data figure 10.**
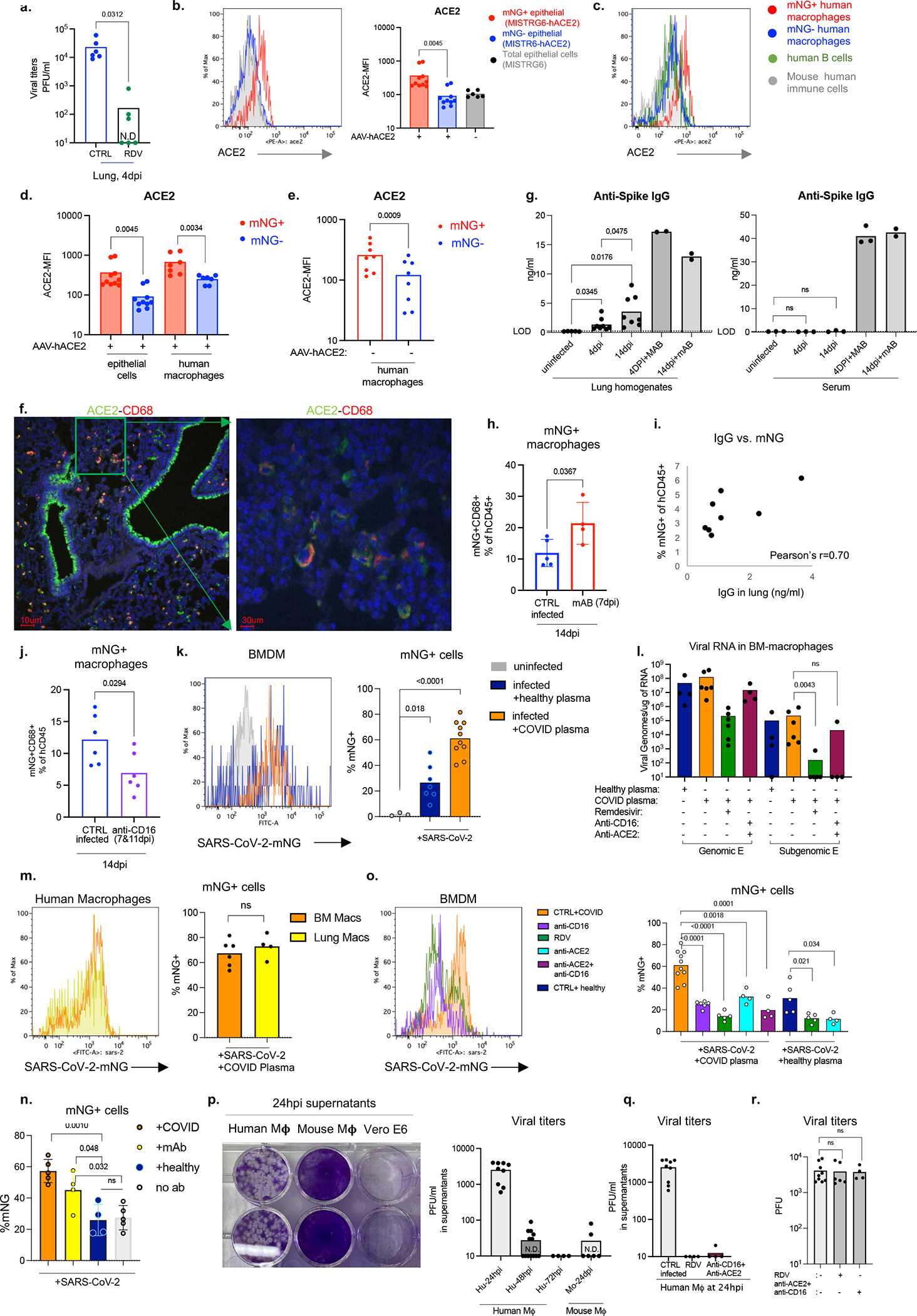
Human lung macrophage infection was enhanced by antibodies and reduced by CD16, ACE2, or RdRp blockade *in vivo* and *in vitro* (matched to figure 2). a. Viral titers in lung homogenates of Remdesivir (RDV) treated or control untreated MISTRG6-hACE2 mice infected with SARS-CoV-2-mNG. CTRL infected n=6, RDV treated n=6 biologically independent mice examined over 3 independent experiments. b. Representative histograms and mean florescent intensity (MFI) for ACE2 expression in mNG+ or mNG− epithelial cells from MISTRG6-hACE2 mice or total epithelial cells from MISTRG6 (AAV−) mice infected with SARS-CoV-2-mNG. AAV+ N=10, AAV− N=6 biologically independent mice examined over at least 3 independent experiments. Paired, two-tailed t-test. c. Representative histograms for ACE2 expression in mNG+ or mNG− human macrophages, human B cells (CD19+) or mouse immune cells isolated from MISTRG6-hACE2 mice infected with SARS-CoV-2-mNG. Representative of N=10 for epithelial cells, N=7 for human macrophages biologically independent mice examined over at least 3 independent experiments. d. MFI of ACE2 expression in mNG+ or mNG− human macrophages or mouse epithelial cells isolated from SARS-CoV-2-mNG infected MISTRG6-hACE2 mice. Epithelial cells n=10, human macrophages n=7 biologically independent mice examined over at least 3 independent experiments. Paired, two-tailed t-test. e. MFI of ACE2 expression in mNG+ or mNG− human macrophages isolated from MISTRG6 (AAV-) mice infected with SARS-CoV-2-mNG. Epithelial cells are virtually not infected with SARS-CoV-2-mNG in MISTRG6 mice without transduced hACE2. N=8 biologically independent mice examined over at least 3 independent experiments. Paired, two-tailed t-test. f. Representative fluorescent microscopy images showing colocalization of human ACE2 and human CD68 cells in SARS-CoV-2 infected MISTRG6-hACE2 mice. Representative of 3 independent mice over 2 independent experiments. g. Anti-Spike (RBD) IgG levels measured by ELISA in serum or lung homogenates of SARS-CoV-2 infected (4 and 14dpi) or uninfected MISTRG6-hACE2 mice treated therapeutically with mAbs (treated at 35hpi or 7dpi) or not. Lung homogenates: Uninfected n=5, 4dpi n=8, 14dpi n=8, 4dpi+mAB n=2, 14dpi+mAB n=2 biologically independent mice representative of at least 2 experiments. Serum: Uninfected n=3, 4dpi n=3, 14dpi n=3, 4dpi+mAB n=3, 14dpi+mAB n=2 biologically independent mice representative of at least 2 experiments. Unpaired, two-tailed t-test. h. Frequencies of mNG signal in human immune cells in infected mice (14dpi) treated therapeutically with monoclonal antibodies^45,64^ (mAb) at 7dpi. CTRL infected n=5, mAb treated n=4 biologically independent mice examined over 2 independent experiments. Means with datapoints and SD. Paired, two-tailed t-test. i. Two-way plot showing anti-Spike (RBD) IgG levels and corresponding mNG+ human immune cell proportions in lungs of infected MISTRG6-hACE2 mice at 4dpi. Pearson’s correlation value =0.70. N=8 biologically independent mice examined over 4 independent experiments. j. Frequencies of mNG+ human macrophages in human immune cells in SARS-CoV-2-mNG infected MISTRG6-hACE2 mice treated with anti-CD16 antibody (Abcam-clone SP175) at 7dpi and 11 dpi and analyzed at 14dpi. n=6 biologically independent mice examined over 3 independent experiments. Unpaired, two-tailed t-test. k. Representative histograms and frequencies of mNG+ cells in BMDMs cultured (or not) with SARS-CoV-2-mNG for 48 hours. Cells were treated with pooled plasma from healthy controls (prior to COVID-19 pandemic) or convalescent COVID-19^45^ patients. Uninfected n=3, infected+ healthy plasma n=7, infected+ COVID plasma n=10 independent samples cultured and analyzed over at least 3 experiments. Means with datapoints. Unpaired t-test. P<0.0001=1.57×10^−5^. l. Quantification of genomic (gRNA) and subgenomic (sgRNA) viral RNA (E gene) in infected BMDMs at 48hpi. Cells were treated with plasma from healthy controls or convalescent COVID-19 patients. Healthy plasma: n=4, COVID plasma n=6, RDV: n=6, anti-CD16+anti-ACE2 n=4 independent samples analyzed over at least 2 independent experiments. Means with datapoints. Mann-Whitney, two-tailed, t-test. m. Representative histograms and frequencies of mNG+ cells in BMDMs and lung macrophages cultured with SARS-CoV-2 in presence of plasma of convalescent COVID-19 patients. mNG+ macrophages were pre-gated on live (live-dead stain negative) cells at 48hpi. BMDMs N=6, Lung macrophages N=4 independent samples analyzed over 2 independent experiments. Unpaired, two-tailed t-test. n. Frequencies of mNG+ cells in BMDMs cultured with SARS-CoV-2 or not in presence of healthy patient plasma, COVID plasma, monoclonal antibodies (clones 135 and 144) or no antibodies. COVID plasma n=5, mAb n=4, healthy plasma n=4, no Ab n=5 independent samples analyzed over 2 independent experiments. Means with datapoints and SD. The same monoclonal antibody cocktail used was used in vivo (Fig. 3). Unpaired, two-tailed t-test. o. Representative histograms and frequencies of mNG+ cells in BMDMs cultured with SARS-CoV-2-mNG (or not) in presence or absence of COVID plasma. Cultures were treated with Remdesivir, anti-human CD16 antibody and/or anti-human ACE2 antibody. Healthy plasma n=5, COVID plasma n=10, RDV n=5, anti-CD16 n=6, anti-ACE2 n=4, anti-CD16+ACE2 n=4 independent samples analyzed over at least 2 independent experiments. Means with datapoints. Unpaired two-tailed t-test. P values<0.0001: anti-CD16 vs COVID plasma= 1.98×10^−5^, RDV vs. COVID plasma= 5.24×10^−6^. p. Viral titers and representative plaque images from supernatants of human or mouse BMDMs infected with SARS-CoV-2 mNG in vitro (without COVID plasma). Infectious virus from supernatants of infected macrophage cultures collected at 24hpi, 48hpi and 72 hpi was plaqued using Vero ACE2+TMPRSS2+ cells. Supernatant collected from Vero E6 cell cultures were provided as reference. Human: 24hpi n=9, 48 hpi n=13, 72hpi n=4. Mouse: 24hpi n=6 independent samples analyzed over at least 2 independent experiments. q. Viral titers from supernatants of BMDMs infected with SARS-CoV-2 mNG in vitro and treated with Remdesivir (RDV) or a combination of anti-CD16 and anti-ACE2 antibodies. Cultures were not supplemented with COVID plasma. Infectious virus from supernatants of infected macrophage cultures collected at 24hpi was plaqued using Vero ACE2+TMPRSS2+ cells. CTRL n=9, RDV n=4, anti-CD16 and anti-ACE2 n=4 independent samples representative of 2 independent experiments. Means with datapoints. r. Viral titers measured as PFUs using supernatants containing concentrations of Remdesivir (1μm) or anti-ACE2 (1μg/ml) and anti-CD16 antibodies diluted to (1:10) allow quantification of PFUs at 24hpi from macrophage cultures. Supernatants were applied on Vero ACE2+ TMPRSS2+ cells which were then infected with a matched inoculum of SARS-CoV-2 mNG (10^3^ PFU quantified in Vero-E6 cells) to test carry over effect in plaque quantification. Untreated N=9, RDV N=6, anti-ACE2+anti-CD16 n=4 independent datapoints collected over 3 independent experiments. Means with datapoints. Unpaired, two-tailed, t-test.

**Extended data figure 11.**
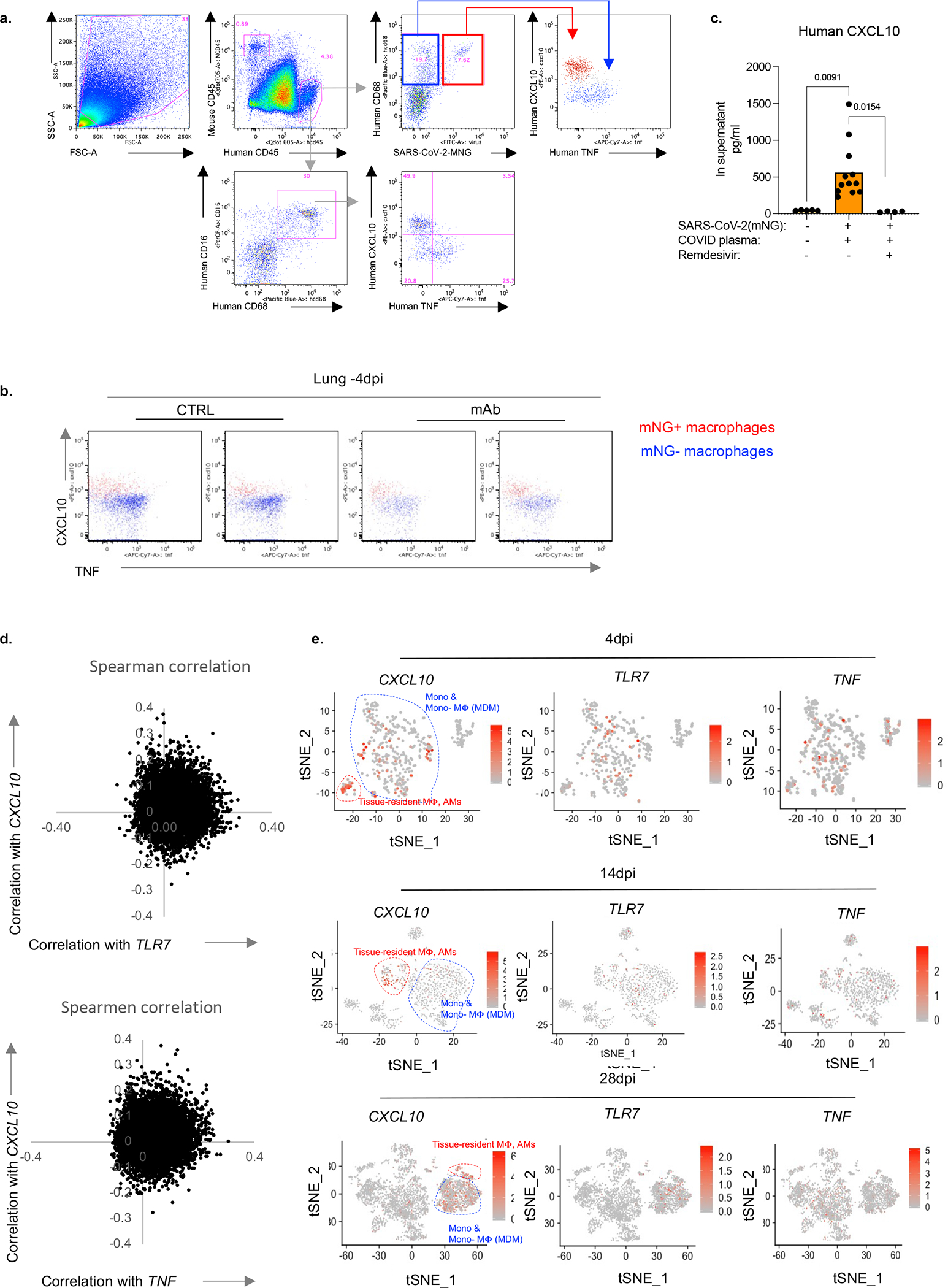
SARS-CoV-2 infected human macrophages have a unique transcriptional signature (matched to figure 3). a. Representative gating strategy of CXCL10 or TNF producing human macrophages in MISTRG6-hACE2 mice infected with SARS-CoV-2-mNG. b. Representative flow cytometry plots of CXCL10 and TNF staining in mice therapeutically treated with mAb or control untreated mice. Representative of n=4 biologically independent mice. C. CXCL10 production measured by ELISA in supernatants of BMDMs infected with SARS- CoV-2 in vitro. Infected BMDM cultures were supplemented with pooled plasma from COVID-19 and were treated with Remdesivir or not. Uninfected n=5, CTRL infected n=12, RDV n=4 over 3 independent experiments. Means with individual values are plotted. Unpaired, two-tailed t-test. c. Spearman correlation values of each gene based on its correlation with *CXCL10* or *TNF* or *TLR7*. d. Expression and distribution of *CXCL10, TNF* and *TLR7* in human immune cells from infected (4, 14 and 28dpi) MISTRG6-hACE2 mice.

**Extended data figure 12.**
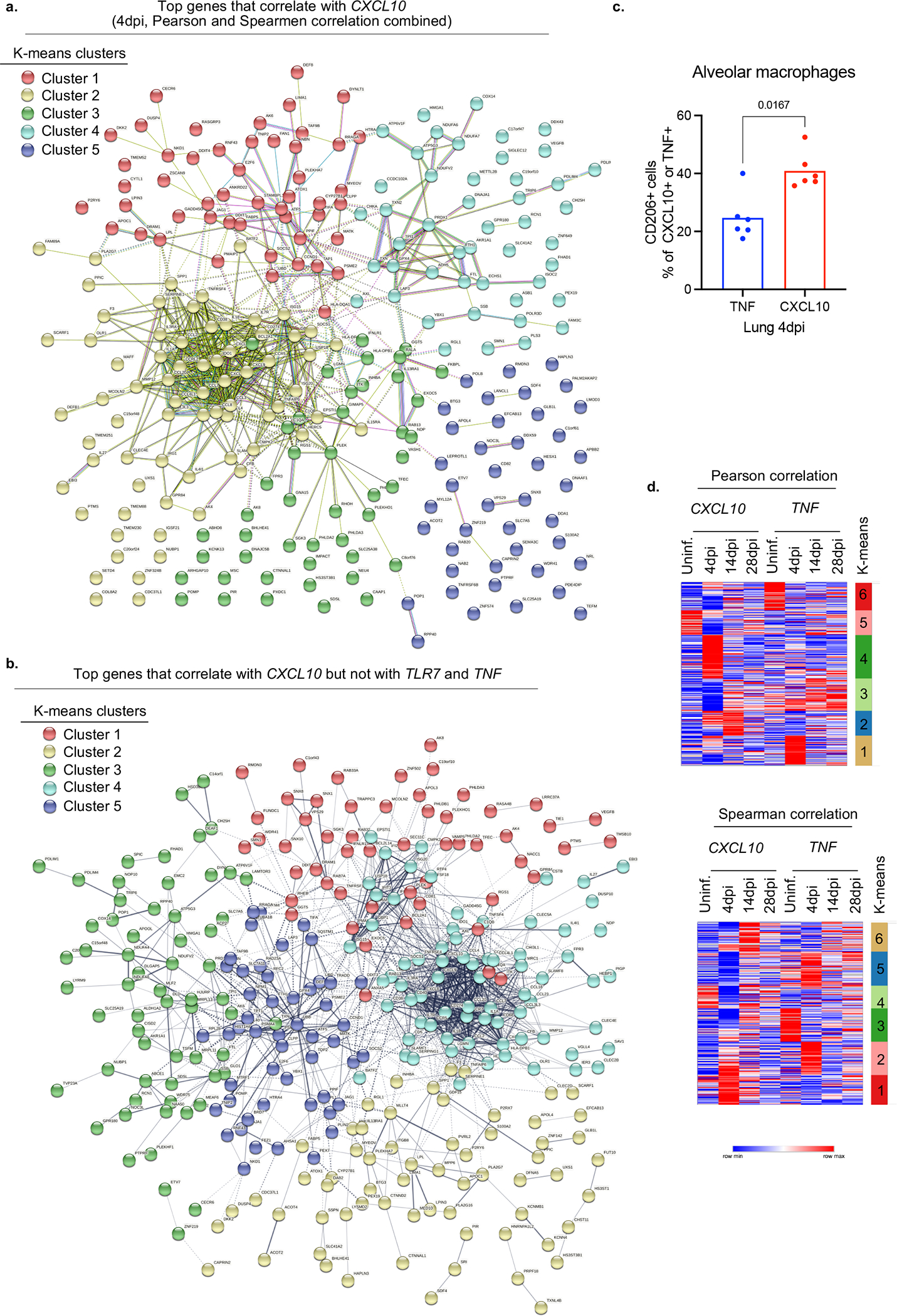
CXCL10-associated genes(matched to figure 3). a. Network (STRING v11.0) analysis of top *CXCL10*-associated genes (top 200 genes). K-means clustering. Clusters and their corresponding pathway analysis are available as source files. Top genes that correlate with CXCL10 (4dpi, Pearson and Spearmen correlation combined) are enriched for distinct inflammatory molecules. b. Network (STRING) analysis of genes that are preferentially associated with CXCL10 but not with TLR7 or TNF. Disconnected nodes in the network are not displayed. K-means clustering. Clusters and their corresponding pathway analysis are presented as source files. c. Proportions of TNF or CXCL10 producing macrophages among alveolar (CD206^hi^CD68^+^) macrophages. Unpaired, two-tailed t-test. N=6 biologically independent mice examined over 3 independent experiments. MISTRG6-hACE2 mice were infected with SARS-CoV-2-mNG and lungs were analyzed at 4dpi. d. Distribution of *CXCL10* or *TNF* associated genes at 4, 14, 28 dpi in lungs infected with SARS-CoV-2 or not. Analysis performed on macrophages of 4dpi lungs in Fig 3d was extended to more timepoints. Pearson (right) and Spearman (left) correlation values were calculated for each gene for its correlation with *CXCL10* or *TNF* in human monocytes and macrophages isolated from uninfected and infected (4, 14 and 28 dpi) lungs. K-means clustering analysis.

**Extended data figure 13.**
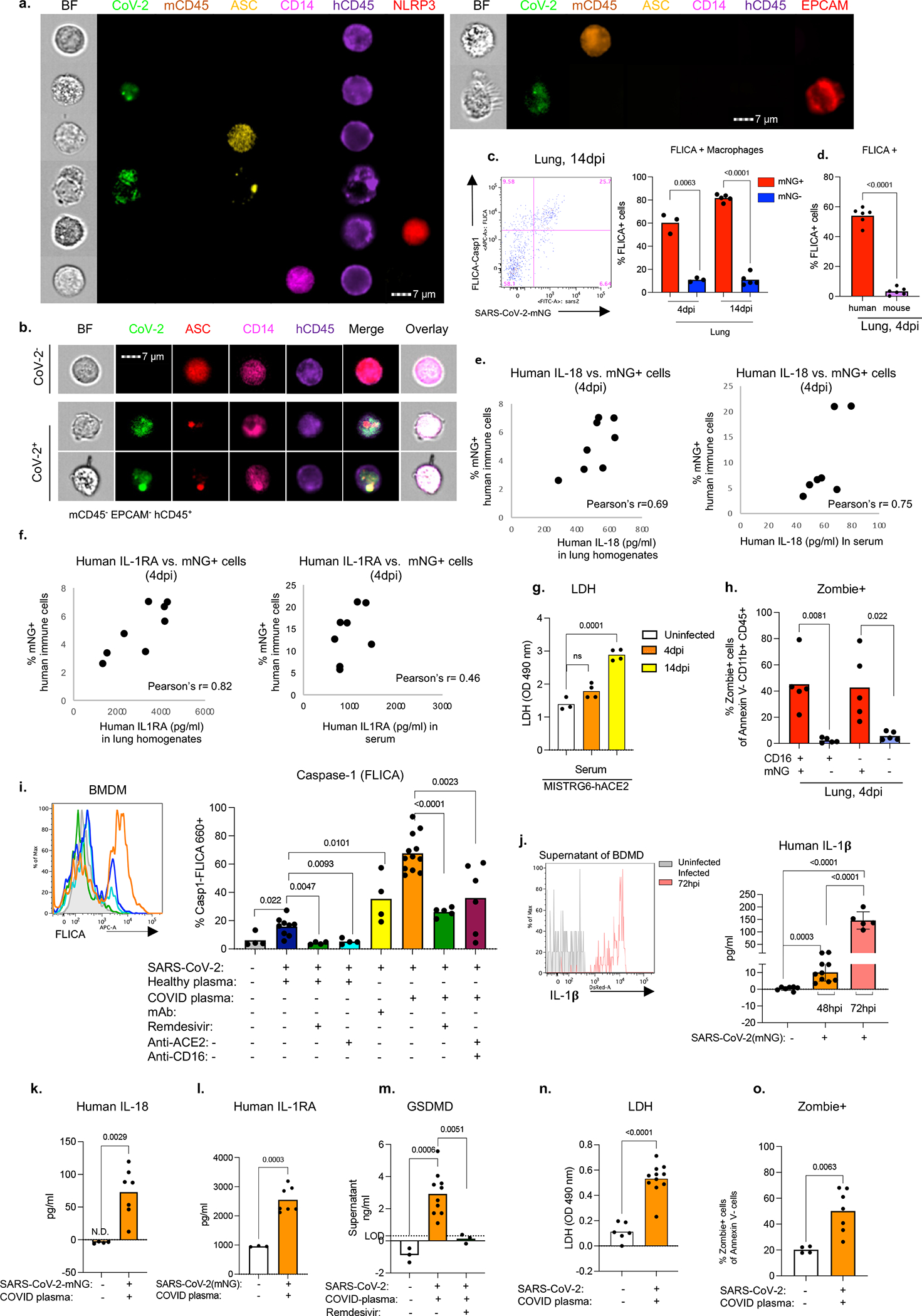
SARS-CoV-2 infection of human macrophages activates inflammasomes and leads to death by pyroptosis *in vivo* and *in vitro* (matched to figure 3). a. Representative images of single stained cells for ASC specks, NLRP3, CD14, human CD45, mouse CD45 and mouse EPCAM. Cells from SARS-CoV-2 infected humanized mice were sorted based on (Extended data Fig. 6j): human immune cells (hCD45^+^); mouse immune cells (mCD45^+^) or epithelial mouse cells (EPCAM^+^). Sorted cells were stained with single antibodies against ASC, CD14 or NLRP3. Left panel shows human immune cells and right panel shows mouse immune cell (mCD45^+^) and mouse epithelial cell (EPCAM^+^). Representative of n=5 independent mouse examined over 3 independent experiments. b. Visualization of ASC specks as a measure of inflammasome activation in mNG+ (SARS-Cov2+) or mNG− (SARS-Cov2−) human immune cells at 4dpi. Human immune cells were sorted from SARS-CoV-2 infected humanized mice were sorted based on expression of human CD45 and mNG and lack of mouse CD45 and EPCAM expression (Extended data Fig. 6j). Representative of n=5 biologically independent mice examined over 3 independent experiments. c. Left: Representative flow cytometry plot displaying SARS-CoV-2-mNG and Casp1-FLICA staining of CD11b^+^ human immune cells. Right: quantification of FLICA^+^ cells (%) as a measure of active caspase-1 in infected (mNG+) and uninfected (mNG−) human lung macrophages (CD11b^+^hCD45^+^) at 4dpi and 14dpi. 4dpi: n=3 biologically independent mice examined over 2 independent experiments, 14dpi n=5 biologically independent mice examined over 3 independent experiments. Lung cells were incubated with FLICA-Casp1 substrate for 30 minutes. Means with individual datapoints plotted. Paired, two-tailed t-test. P<0.0001=4.29×10^−9^. d. Quantification of Casp1-FLICA staining as a measure of active caspase-1 in infected (mNG+) human or total mouse CD11b+ cells at 4dpi. Mouse cells: mCD45+CD11b+hCD45−. Human cells: mCD45−CD11b+hCD45+mNG+. N=6 biologically independent mice examined over 3 independent experiments. Means with individual datapoints plotted. Paired, two-tailed t-test. P<0.0001=1.79×10^−9^. e. Human IL-18 (measured by ELISA) in lungs and serum and corresponding mNG levels (measured as percent within human immune cells by flow cytometry) in lungs of infected MISTRG6-hCE2 mice at 4dpi. Lung: Pearson’s correlation value=0.69. N=8 biologically independent mice examined over 3 independent experiments. Serum: Pearson’s correlation value=0.75. n=7 biologically independent mice examined over 3 independent experiments. Unpaired, two-tailed t-test. f. Human IL-1RA (measured by ELISA) in lungs and serum and corresponding mNG levels (measured as percent within human immune cells by flow cytometry) in lungs of infected MISTRG6-hCE2 mice at 4dpi. Lung: Pearson’s correlation value=0.82 n=8 biologically independent mice examined over 3 independent experiments. Serum: Pearson’s correlation value=0.46 n=8 biologically independent mice examined over 3 independent experiments. Unpaired t-test, two-tailed. g. LHD levels measured as absorbance at OD 490nm in serum of uninfected or infected MISTRG6-hACE2 mice at 4dpi and 14dpi. Fresh serum was assayed for LDH. Uninfected n=3, 4dpi n=4, 14dpi n=4 biologically independent mice examined over 2 independent experiments. Means with individual datapoints. Unpaired, two-tailed t-test. h. Zombie Aqua incorporation in infected (mNG^+^) or uninfected (mNG^−^) CD16^+^CD11b^+^ or CD16^−^CD11b^+^ human myeloid cells. Frequencies of Zombie+ cells were measured in Annexin V− cells. N=5 biologically independent mice examined over 2 independent experiments. Means with individual datapoints. Paired, two-tailed t-test. i. Representative histograms and quantification of Casp1-FLICA staining as a measure of active caspase-1 in bone marrow derived macrophages (BMDMs) infected with SARS-CoV-2 in vitro or not for 48 hours. BMDM cultures were either supplemented with healthy or COVID plasma or monoclonal antibodies for the duration of the infection. Cultures were treated with Remdesivir, anti-ACE2 and anti-CD16 to block viral replication or viral entry. Coloring on the histograms matches the bar graph legend. Uninfected n=4; healthy plasma CTRL infected n=9, anti-ACE2 n=4, RDV n=4; mAb n= 4; COVID plasma CTRL infected n=12, anti-ACE2+anti-CD16 n=6, RDV n=5 independent datapoints collected over at least 2 independent experiments. Means with all datapoints. Unpaired, two-tailed t-test. P-values< 0.0001: COVID plasma vs. RDV: 5.85×10^−6^. j. Representative histograms and quantification of IL-1β in supernatants of BMDMss infected with (or not) SARS-CoV-2 in vitro for 48 or 72 hours. Uninfected n=7, 48hpi n=10, 72hpi n=5 independent datapoints collected over 3 independent experiments. Means with SD and individual datapoints. Unpaired, two-tailed t-test. P-values< 0.0001: uninfected vs 72hpi= 4.96×10^−7^, 48hpi vs 72hpi=1.17 ×10^−8^. k. Human IL-18 levels at 48hpi in supernatants of BMDMss infected or not with SARS-CoV-2 in vitro. BMDMss cultures were supplemented with COVID plasma for the duration of the infection. Uninfected n=4, 48hpi n=7 independent datapoints collected over at least 2 independent experiments. Unpaired, two-tailed t-test. Means with individual datapoints. Unpaired, two-tailed t-test. l. Human IL-1RA levels at 48hpi in supernatants of BMDMss infected with SARS-CoV-2 in vitro or not. Uninfected n=3, infected n=7 independent datapoints collected over at least 2 independent experiments. Unpaired, two-tailed t-test. Means with individual datapoints. Unpaired, two-tailed t-test. m. Gasdermin D (GSDMD) levels in supernatants of BMDMss infected with (or not) SARS-CoV-2 *in vitro* for 48 hours. BMDM cultures were supplemented with COVID plasma for the duration of the infection. Cultures were treated with Remdesivir to block viral replication. Uninfected N=3, CTRL infected n=10, RDV n=3 independent datapoints collected over at least 2 independent experiments. Means with individual datapoints. Unpaired, two-tailed t-test. n. LHD levels measured by absorbance at OD 490nm in supernatants of infected or uninfected. Uninfected n=6, Infected n=11 independent datapoints collected over 3 independent experiments. Means with individual datapoints. Unpaired, two-tailed t-test. P-value= 7.38×10^−6^. o. Zombie Aqua incorporation in uninfected or SARS-CoV-2-mNG infected BMDMs. Frequencies of Zombie+ cells within Annexin V− population at 48hpi are presented. Uninfected n=4, infected n=7 independent datapoints collected over 3 independent experiments. Means with individual datapoints. Unpaired, two-tailed t-test.

**Extended data figure 14.**
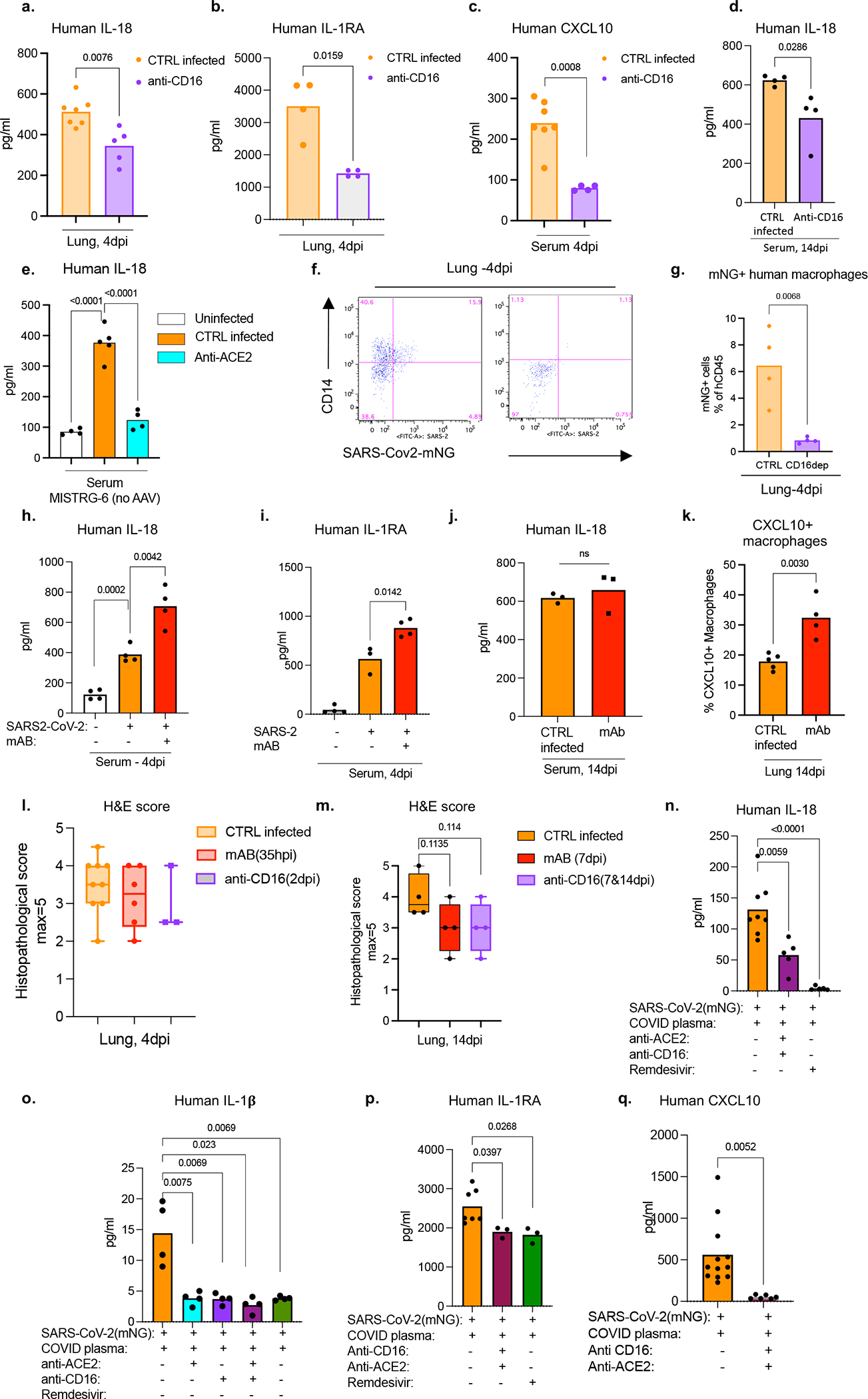
Promoting or blocking viral entry or replication in human macrophages in vivo and in vitro impacts inflammatory profile of macrophages (matched to figure 3). a. Human IL-18 levels measured in lung homogenates of infected (4dpi) MISTRG6-hACE2 mice treated (or not) with CD16 blocking antibody (Abcam). CTRL-infected N=7, ant-CD16 n=5 biologically independent mice examined over 3 independent experiments. Means with datapoints. Unpaired, two-tailed t-test. b. Human IL-1RA levels measured in lung homogenates of infected (4dpi) MISTRG6-hACE2 mice treated (or not) with CD16 blocking antibody (Abcam). N=4 biologically independent mice examined over 2 independent experiments. Means with all datapoints. Unpaired, two-tailed t-test. c. Human CXCL10 levels measured in serum of infected (4dpi) MISTRG6-hACE2 mice treated (or not) with CD16 blocking antibody (Abcam). CTRL infected N=7, anti-CD16 n=4 biologically independent mice examined over at least 2 experiments. Mean with individual values. Unpaired, two-tailed t-test. d. Human IL-18 levels measured in serum of infected (14dpi) MISTRG6-hACE2 mice treated (or not) with CD16 blocking antibody (Abcam). Mice were treated with anti-CD16 blocking antibody at 7dpi and 11dpi. CTRL infected N=4, anti-CD16 n=4 biologically independent mice examined over 2 independent experiments. Means with individual datapoints. Unpaired, two-tailed t-test. e. Human IL-18 levels measured in serum of infected (4dpi) MISTRG6 mice treated (or not) with anti-ACE2 antibody (Abcam). Mice were treated with anti-ACE2 antibody at 1,2,3 dpi. Uninfected n=4, CTRL infected n=5, anti-ACE2 n=4 biologically independent mice examined over 2 independent experiments. Means with individual datapoints. Unpaired, two-tailed t-test. P<0.0001= uninfected vs CTRL-infected=1.43×10^−5^, CTRL-infected vs anti-ACE2=6.95×10^−5^. f. Representative flow cytometry plots of CD14 staining on total human immune cells (hCD45+) as a proxy for myeloid cells in infected MISTRG6-hACE2 mice (4dpi) treated (or not) with a depleting antibody against CD16 (ThermoFisher, clone 3G8). MISTRG6-hACE2 mice were infected with SARS-CoV-2-mNG. Representative of n=4 biologically independent mice examined over 2 independent experiments. g. Frequencies of mNG+ cells in infected MISTRG6-hACE2 mice at 4dpi treated (or not) with a depleting antibody against CD16 (ThermoFisher, clone 3G8). N=4 biologically independent mice examined over 2 independent experiments. Means with datapoints. Unpaired, two-tailed t-test. h. Human IL-18 levels in serum of infected or uninfected MISTRG6-hACE2 mice that were therapeutically treated with monoclonal antibodies (mAb) at 36hpi or not. Sera from infected mice were analyzed at 4dpi. N=4 biologically independent mice examined over 2 independent experiments. Means with datapoints. Unpaired, two-tailed t-test. i. Human IL-1RA levels in serum of infected (4dpi) or uninfected MISTRG6-hACE2 mice that were therapeutically treated with mAb at 36hpi or not. Uninfected and mAB treated N=4, CTRL infected n=3 (matched to mAb treatment) biologically independent mice examined over 2 independent experiments. Means with datapoints. Paired, two-tailed t-test. j. Human IL-18 levels measured in serum of infected (14dpi) MISTRG6-hACE2 mice treated (or not) with mAb (clone 135+ clone 144) at 7dpi and analyzed at 14dpi. N=3 biologically independent mice examined over 2 independent experiments. Mean with individual datapoints. Unpaired, two-tailed t-test. k. Frequencies of CXCL10+ macrophages within total human macrophages (hCD45+hCD68+) in lungs of infected MISTRG6-hACE2 mice treated (or not) with mAb (clone 135, clone 144) at 7dpi and analyzed at 14dpi. Mean with individual values. Unpaired, two-tailed t-test. CTRL-infected N=5, mAb N=4 biologically independent mice examined over 2 independent experiments. l. Box and whisker plot (min to max, with all datapoints) of the histopathological scoring of the H&E staining of infected MISTRG6-hACE2 lungs at 4dpi. Mice were either treated with monoclonal antibodies at 35hpi or anti-CD16 at 2dpi. CTRL infected N=9, mAb treated n=6, anti-CD16 treated n=3 biologically independent mice examined over at least 2 independent experiments. The whiskers go down to the smallest value (minimum) and up to the largest value (maximum). The box extends from the 25th to 75th percentiles. The median is shown as a line in the center of the box. Unpaired t-test, not significant. m. Box and whisker plot (min to max, with all datapoints) of the histopathological scoring of the H&E staining of infected MISTRG6-hACE2 lungs at 14dpi. Mice were either treated with monoclonal antibodies at 7dpi or anti-CD16 at 7 and 11dpi. CTRL infected N=4, mAb treated n=4, anti-CD16 treated n=4 biologically independent mice examined over 2 independent experiments. The whiskers go down to the smallest value (minimum) and up to the largest value (maximum). The box extends from the 25th to 75th percentiles. The median is shown as a line in the center of the box. Unpaired, two-tailed t-test, not significant. n. Human IL-18 levels in supernatants of SARS-CoV-2 infected BMDMs treated with anti-CD16 and anti-ACE2 antibodies to block viral entry or with Remdesivir to block viral replication. CTRL infected n=8, anti-CD16+anti-ACE2 n=5, RDV n=5 independent datapoints over 3 independent experiments. Means with all datapoints. Unpaired, two-tailed t-test. P<0.0001= 5.0×10^−5^. o. Human IL-1β levels in supernatants of SARS-CoV-2 infected BMDMs treated with anti-CD16 and anti-ACE2 antibodies to block viral entry or with Remdesivir to block viral replication. N=4 independent datapoints over 2 independent experiments. Means with all datapoints. Unpaired, two-tailed, t-test. p. Human IL-1RA in supernatants of SARS-CoV-2 infected BMDMs treated with anti-CD16 and anti-ACE2 antibodies to block viral entry or with Remdesivir to block viral replication. CTRL infected n=7, anti-CD16+anti-ACE2 N=3, RDV n=3 independent datapoints over 2 independent experiments. Means with all datapoints. Unpaired, two-tailed, t-test. q. Human CXCL10 levels in supernatants of BMDMs infected *in vitro* in presence or absence of anti-CD16 and anti-ACE2 antibodies to block viral entry. CTRL infected N=12, anti-ACE2+ anti-CD16 treated n=6 independent datapoints over 2 independent experiments. Means with all datapoints. Unpaired, two-tailed, t-test.

**Extended data figure 15.**
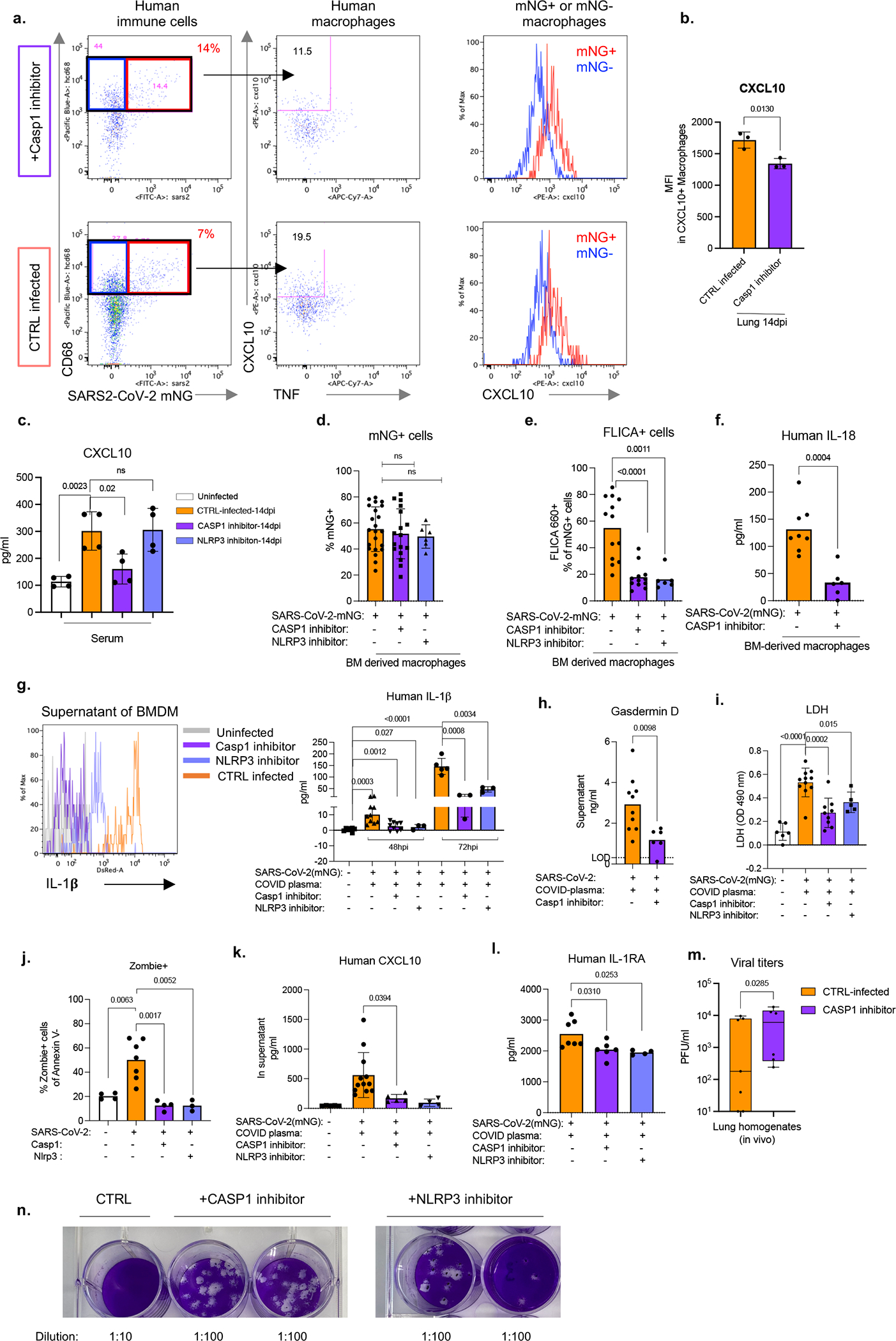
Blockade of inflammasome activation leads to reduced cytokine production in vitro (matched to figure 4). a. Representative flow cytometry plots of CXCL10 and TNF staining in total macrophages and histograms of CXCL10 expression in infected (mNG^+^) and uninfected (mNG^−^) macrophages from lungs of SARS-CoV-2-mNG infected MISTRG6-hACE2 mice treated with caspase-1 (Casp1) or NLRP3 inhibitors in vivo. Mice were treated on days 6,8,10,12 post-infection and analyzed at 14dpi. Representative of n=5 biologically independent mice. b. Mean fluorescent intensity (MFI) of CXCL10 expression in human macrophages isolated from infected MISTRG6-hACE2 mice treated with Casp1 inhibitor or left untreated. N=3 biologically independent mice. Representative of 3 independent experiments. Means with all datapoints and SD. Unpaired, two-tailed t-test. c. CXCL10 levels in serum of SARS-CoV-2-mNG infected MISTRG6-hACE2 mice (14dpi) treated with Casp1 or NLRP3 inhibitors. n=4 biologically independent mice examined over 2 independent experiments. Means with all datapoints and SD. Unpaired, two-tailed t-test. d. Frequencies of mNG^+^ bone marrow-derived macrophages (BMDMs) infected with SARS-CoV-2-mNG in vitro. BMDMs were treated with caspase-1 (Casp1) or NLRP3 inhibitors or left untreated and analyzed at 48hpi. CTRL infected n=22, Casp1 inhibitor n=17, NLRP3 inhibitor n=6 independent datapoints collected over at least 3-experiments. Means with all datapoints and SD. Unpaired, two-tailed t-test. e. Frequencies of FLICA^+^ BMDMs infected with SARS-CoV-2 in vitro for 48 hours. BMDMs were treated with Casp1 or NLRP3 inhibitors or left untreated. CTRL infected n=13, Casp1 inhibitor n=12, NLRP3 inhibitor n=6 independent datapoints collected over at least 3 independent experiments. Means with all datapoints. Unpaired, two-tailed t-test. P<0.0001=3.33×10^−5^. f. Human IL-18 levels in supernatants of SARS-CoV-2-mNG infected BMDMs treated with caspase-1 (Casp1) inhibitor or left untreated. CTRL infected n=8, Casp1 inhibitor-treated n=6 independent datapoints collected over 2 independent experiments. Means with all datapoints and SD. Unpaired, two-tailed t-test. g. Representative histograms and quantification of IL-1β in supernatants of BMDMs infected with SARS-CoV-2 in vitro. Cultures were treated with caspase-1 (Casp1) inhibitor. over at least 2 independent experiments. Uninfected n=7; 48hpi CTRL infected n=10, Casp1 inhibitor n=9, NLRP3 inhibitor n=3; 72hpi CTRL infected n=5 Casp1 inhibitor n=3, NLRP3 inhibitor n=3 independent datapoints collected over at least 2 experiments. Means with all datapoints and SD. Unpaired, two-tailed t-test. P<0.0001=4.96×10^−7^. h. Human Gasdermin D (GSDMD) levels at 48hpi in supernatants of SARS-CoV-2-mNG infected BMDMs treated with Casp1 inhibitor or left untreated. CTRL infected n=10, Casp1 inhibitor-treated n=6 independent datapoints collected over at least 3 independent experiments. Means with all datapoints. Unpaired, two-tailed t-test. i. LDH levels measured as absorbance at OD 490nm in supernatants of uninfected or SARS-CoV-2-mNG infected BMDMs treated with Casp1 or NLRP3 inhibitor or left untreated in vitro. Uninfected n=6, CTRL infected (48hpi) n=11, Casp1 inhibitor-treated(48hpi) n=9, NLRP3 inhibitor-treated (48hpi) n=5 independent datapoints collected over 2 independent experiments. Means with all datapoints and SD. Unpaired, two-tailed t-test. P<0.0001=7.38×10^−6^. j. Zombie Aqua incorporation in SARS-CoV-2-mNG infected BMDMs treated with Casp1 or NLRP3 inhibitor or left untreated (CTRL infected). Frequencies of Zombie+ cells within Annexin V− population at 48hpi are reported. Uninfected n=4, CTRL infected n=7, Casp1 inhibitor n=4, NLRP3 inhibitor n=3 over 2 experiments. Means with all datapoints. Unpaired, two-tailed t-test. k. Human CXCL10 levels in supernatants of infected human BMDMs treated with Casp1 inhibitor or NLRP3 inhibitor or left untreated. Supernatants were collected at 48hpi. Uninfected n=5, CTRL infected n=12, Casp1 inhibitor n=5, NLRP3 n=4 independent datapoints over at least 2 independent experiments. Means with all datapoints and SD. Unpaired, two-tailed t-test. l. Human IL-1RA levels in supernatants of SARS-CoV-2-mNG infected human BMDMs treated with Casp1 inhibitor or not. BMDMs were treated with Casp1 inhibitor. Supernatants were collected at 48hpi. CTRL infected n=7, Casp1 inhibitor-treated n=6, NLRP3 inhibitor-treated n=4 independent datapoints collected over at least 2 independent experiments. Means with all datapoints. Unpaired, two-tailed t-test. m. Viral titers measured as PFU in lung homogenates of MISTRG6-hACE2 mice infected with SARS-CoV-2 and treated with caspase-1 inhibitor in vivo. Infected MISTRG6-hACE2 mice were treated with Caspase 1 inhibitor on days 6,8,10,12 post-infection and analyzed at 14dpi. Lung homogenates were plaqued using Vero ACE2+TMPRSS2+ cells. of CTRL infected: n=7, Casp1 inhibitor-treated: n=6 biologically independent mice examined over 3 independent experiments. Box and whisker plot (min to max, with all datapoints) The whiskers go down to the smallest value (minimum) and up to the largest value (maximum). The box extends from the 25th to 75th percentiles. The median is shown as a line in the center of the box. Ratio paired, two-tailed t-test. n. Representative images of plaque assays used to quantify infectious virus in supernatants of BMDMs infected with SARS-CoV-2-mNG and treated with caspase-1 or NLRP3 inhibitors. Supernatants of infected macrophage cultures were collected at 48hpi and plaqued using Vero ACE2^+^TMPRSS2^+^ cells. Plaques were resolved at 48hpi. Representative of CTRL infected: n=13, Casp1 inhibitor-treated: n=8, NLRP3 inhibitor-treated: n=5 independent datapoints collected over 3 independent experiments.

## Supplementary Material

Supplementary Table 1

Supplementary Table 2

Supplementary Table 4

Supplementary Table 5

Supplementary Discussion

Supplementary Table 3
