# Supplementary material for: Inflammasome activation in infected macrophages drives COVID-19 pathology
Source: Nature. Author manuscript; Available in PMC 2022 Aug 1. (PMC9288243; doi:10.1038/s41586-022-04802-1)
Supplement: Supplementary Table 5 [file NIHMS1809392-supplement-Supplementary_Table_5.pdf]

Supplementary Table 5. Patient specimens used for immunofluorescent (IF) staining.

Case	Age	Gender	Medication	Death (dps)	Co-morbidities	Cause of death	Histopathological findings
COVID sample#1	54	male	Dexamethasone, Remdesivir	20	Obese	Acute fibrinous organizing pneumonia	Diffuse alveolar damage, overlapping features of exudative and proliferative phase
COVID sample#2	37	male		35	alcoholic liver disease and micro nodular cirrhosis.	Complications of COVID-19	Diffuse alveolar damage, overlapping features of exudative and proliferative phase
COVID sample#3	77	female	Dexamethasone, Remdesivir	32	Diabetes, Chronic Kidney disease, COPD Obesity	Complications of COVID-19	Diffuse alveolar damage, overlapping features of exudative and proliferative phase
COVID sample#4	74	male		25	Diabetes, Dementia, Coronary artery disease Hyperlipidemia,	Complications of COVID-19	Diffuse alveolar damage, predominantly proliferative phase
Non-COVID control #1	74	male	n/a	n/a	Chronic obstructive pulmonary disease	Chronic obstructive pulmonary disease and pneumonia	Pneumonia
Non-COVID control #2	67	male	n/a	n/a	Myelodysplastic syndrome	Pneumonia	Diffuse fibrinous and organizing pneumonia
Non-COVID control # 3	50	male	n/a	n/a	Chronic kidney disease, sarcoidosis, chronic pancreatitis	Cryptococcal pneumonia	Cryptococcal pneumonia
