# Supplementary material for: Inflammasome activation in infected macrophages drives COVID-19 pathology
Source: Nature. Author manuscript; Available in PMC 2022 Aug 1. (PMC9288243; doi:10.1038/s41586-022-04802-1)
Supplement: Supplementary Discussion [file NIHMS1809392-supplement-Supplementary_Discussion.pdf]

Supplementary Materials

Inflammasome activation in infected macrophages drives COVID-19 pathology

Correspondence to: richard.flavell@yale.edu

Esen Sefik[1], Rihao Qu[1,2,13], Caroline Junqueira[3,4,5] Eleanna Kaffe[1], Haris Mirza[1,2], Jun Zhao[1,2], J. Richard Brewer[1], Ailin Han[1], Holly R. Steach[1], Benjamin Israelow[1], Holly N. Blackburn[1,10], Sofia E. Velazquez[1], Y. Grace Chen[1], Stephanie Halene[2,6], Akiko Iwasaki[1,9], Eric Meffre[1], Michel Nussenzweig[7,10], Judy Lieberman[3,4], Craig B. Wilen[1, 8,] Yuval Kluger[2,12], Richard A. Flavell[1,9]

Author information:

1. Department of Immunobiology, Yale University School of Medicine, New Haven, CT, USA.
2. Department of Pathology, Yale University School of Medicine, New Haven, CT, USA.
3. Program in Cellular and Molecular Medicine, Boston Children's Hospital, Boston, MA, USA.
4. Department of Pediatrics, Harvard Medical School, Boston, MA, USA.
5. Instituto René Rachou, Fundação Oswaldo Cruz, Belo Horizonte, Minas Gerais, Brazil.
6. Section of Hematology, Yale Cancer Center and Department of Internal Medicine, Yale University School of Medicine, New Haven, CT.
7. Laboratory of Molecular Immunology, The Rockefeller University, New York, NY, USA.
8. Department of Laboratory Medicine, Yale University School of Medicine, New Haven, CT, USA.
9. Howard Hughes Medical Institute, Yale University School of Medicine, New Haven, CT, USA.
10. Howard Hughes Medical Institute, The Rockefeller University, New York, NY, USA.
11. Department of Surgery, Yale University School of Medicine, New Haven, CT, USA.
12. Program of Applied Mathematics, Yale University, New Haven, CT, USA
13. Computational Biology & Bioinformatics Program, Yale University, New Haven, CT, USA

Contents

Supplementary Discussion 2

Infection of human macrophages and inflammasome activation 2

Mechanisms of viral uptake 2

Infected human macrophages initiate and maintain an inflammatory cascade that impacts disease outcome 4

Viral sensing by NLRP3 in infected human macrophages 5

Infiltrating macrophages and the essential role of viral RNA-dependent type I IFN response in disease pathology 6

Implications of our findings 6

Supplementary Tables 7

Table S1: Human genes that are differentially regulated in lungs of infected MISTRG6-hACE2 in response to therapeutics. 7

Table S2: Cluster identifying markers and markers that identify temporal transcriptional changes associated with monocytes and macrophages in infected (4, 14 or 28dpi) or uninfected lungs of MISTRG6-hACE2 mice (matched to Fig 1g). 7

Table S3: Expression of human genes that are enriched in macrophages (clusters identified as part of Fig. 1g) during SARS-Cov-2 infection and their response to anti-IFNAR2 and Remdesivir therapy (matched to Fig. 1h). Normalized expression of duplicates analyzed. 7

Table S4: Pearson and spearman correlation values calculated for each gene for its correlation with CXCL10, TNF or TLR7 in human monocytes and macrophages at 4dpi (based on Fig. 1g, matched to Fig. 3d). 7

Table S5: Patient specimens used for immunofluorescent (IF) staining. 7

Supplementary References: 8

Supplementary Discussion

Infection of human macrophages and inflammasome activation

The MISTRG6 model of COVID-19 faithfully reflects many of the chronic immunoinflammatory features of the human disease and provides an opportunity to dissect the mechanisms of late immunopathogenesis in this disease[19]. As in severe human disease, COVID-19 in MISTRG6-hACE2 mice presents with persistent viral RNA, chronic IFN response accompanied with a chronic inflammatory state in macrophages that is initiated by infection of human macrophages and maintained by subsequent inflammasome activation[19]. These events may eventually contribute to the development of persistent pulmonary immunopathology and fibrosis, which is supported by histopathological and transcriptional analysis of lungs late in infection. Overall, our mechanistic study of this model defines a cascade of events, which, initiates with lung epithelial infection and is followed with infection of tissue-resident macrophages in an ACE2 and CD16 mediated manner. SARS-CoV-2 replicates in these macrophages generating replicative intermediates which include dsRNA, subgenomic viral RNA, viral RNA polymerase (RdRp), and expression of a virally encoded fluorescent reporter gene (mNG), all of which is inhibited by Remdesivir, an inhibitor of viral replication. SARS-CoV-2 replication and replicative intermediates activate an inflammatory program which involves activation of inflammasomes, production, and release of inflammatory cytokines and chemokines, and finally pyroptosis. We established inflammasome activation by visualizing ASC speck formation, which colocalized with active caspase-1 and NLRP3; this led to maturation of inflammasome mediated cytokines IL-1β and IL-18, and results in pyroptosis assayed by gasdermin D (GSDMD) and LDH release. Inflammasome activation and downstream effectors in these infected macrophages are caspase-1 and NLRP3 dependent, as inhibitors of both caspase-1 and at NLRP3 block all downstream aspects of inflammasome activation and the inflammatory cascade both *in vivo* and *in vitro*. More importantly, targeting inflammasome mediated hyperinflammation prevented immunopathology associated with chronic SARS-CoV-2 infection *in vivo*.

Mechanisms of viral uptake

Consistent with the enhancing role for antiviral antibodies in macrophage infection, COVID-19 severity in patients was correlated with early, high levels of afucosylated IgG which enhanced the inflammatory response by monocytes and macrophages through Fc-mediated interactions with CD32 and CD16[47-49]. We observe a similar role for CD16 and antibodies in humanized mice infected with SARS-CoV-2-mNG. The frequency of infected macrophages which express high levels of CD16 correlated with the levels of anti-Spike antibodies in the lung tissue, particularly at 4dpi. mNG positivity in these cells also

correlated with a strong proinflammatory cytokine signature as measured by elevated levels of IL-18, IL-1RA, and CXCL10, all of which contribute to severe disease in humans. CD16 blockade *in vivo* and *in vitro* prevented viral uptake and blocked this subsequent inflammatory response as measured by reduced levels of CXCL10, IL-18, and IL-1RA.

The ACE2 receptor that is utilized by SARS-CoV-2 to infect lung epithelium is also expressed preferentially by infected human macrophages *in vivo*[43]. Notably, $CD14^{hi}CD16^{hi}$ cells and alveolar macrophages which had measurable viral RNA cells in patient samples did not appear to co-express the traditional viral entry factors, ACE2 and TMPRSS2 as measured by the relatively insensitive method of single cell RNA sequencing (scRNAseq)[7]. This may however be a technical limitation as we similarly could not detect measurable ACE2 transcript in alveolar macrophages or CXCL10+ macrophages (which is a proxy for mNG+ infected macrophages) by scRNAseq at any time point during infection. Yet, ACE2 protein clearly colocalized with CD68, a marker of human macrophages and correlated with viral replication quantified by mNG in these cells. More importantly blocking ACE2 prevented viral uptake by macrophages. Interestingly in our system, *ACE2* expression was inducible (data not shown, GSE186794), and its levels, correlated well with normalized viral RNA levels measured in the same samples (data not shown, GSE186794). To determine factors that could regulate ACE2 expression, we identified genes that correlate with *ACE2*. Interestingly, the top 100 genes that correlate with *ACE2* expression (r>0.6) were enriched for interferon responsive genes (data not shown, GSE186794), further highlighting the importance of the interferon pathway in COVID19 patients.

Infection of macrophages in our system is therefore dependent on both ACE2 receptor as well as antibody-mediated uptake by CD16. Given the prevalence of antibodies increases as the disease progresses, it is likely that the latter mechanism plays a more important role later in infection. However, there may be other mechanisms that enhance SARS-CoV-2 infection or the downstream inflammatory response in human macrophages that are not explored in this study. SARS-CoV-2-mNG lacks-Orf7a which can enhance proinflammatory cytokine production in monocytes via its interaction with CD14[41,77]. It is not clear whether CD14 expression in macrophages could mediate viral entry or enhance inflammatory cytokine production[77]. It has been noted that low molecular weight immune complexes formed prior to the specific humoral response, combined with the afucosylated state of IgGs, can further enhance the CD16-mediated activation of monocytes and macrophages, mimicking a state similar to systemic lupus erythematosus (SLE) disease. Given that we observe SLE-like features in our lung transcriptome[19], it is possible that blocking CD16-mediated macrophage infection and activation may impact this SLE-like state observed late in our disease model.

Infected human macrophages initiate and maintain an inflammatory cascade that impacts disease outcome

In our mouse model, monocytes, and macrophages in SARS-CoV-2 infected MISTRG6-hACE2 are central to disease pathology and are the main source of inflammatory cytokines IL-1β, IL-1RA, IL-18, TNF-α, IL6 and inflammatory chemokines like CXCL10. It is likely that this is also true in human disease[7,15,20,78]. Of these cytokines and chemokines elevated in COVID-19 patients, IL-1β, IL-1RA, IL-18 and CXCL10 also correlated with disease severity[2-7,14,15,20,79]. Infected macrophages in MISTRG6-hACE2 mice (and *in vitro* infected BMDM) were the main producers of these cytokines correlating with disease severity and had a unique transcriptional signature revealed by association with *CXCL10* correlating transcripts in our transcriptional datasets. In humans, SARS-CoV-2 viral RNA was detected in mononuclear phagocytes characterized by scRNA-seq analysis of autopsied lungs of COVID-19 patients[7,20] although whether this results from viral replication in these cells or phagocytosis could not be distinguished. In line with our findings, $CD14^{hi}CD16^{hi}$ cells and alveolar macrophages in autopsied lungs of COVID-19 patients were particularly enriched with viral RNA[7,20]. We also found clear evidence for the presence of viral components Including viral RdRp in macrophages and epithelial cells of infected human lungs. Several recent studies of human macrophages and other myeloid cells also suggest that SARS-CoV-2 can infect these cells[80-82]. However, in our humanized mouse system it is clear that the majority of RNA found associated with host cells may be the result of phagocytic or other non-replicative uptake mechanisms. It was only by using SARS-CoV-2-mNG virus (see Fig. 2, 3) that we were able to distinguish these two processes at which point we could clearly distinguish infection from mere uptake of viral debris, which in fact is prevalent. Some SARS-CoV-2+ myeloid cells in humans also had distinct transcriptomes which were largely recapitulated in what we construe as *CXCL10* associated genes (*CXCL11, CCL18, CCL8, ISG15, CD83;* Fig. 3) from MISTRG6-hACE2, with the exception of TNF which was co-expressed by these same CXCL10+ cells. This in fact is complementary to our findings where the CXCL10-associated gene signature and its inverse relationship with TNF weakens late in infection (28dpi, Extended data figure 12d), a time point that corresponds to autopsied lungs of severe COVID-19, suggesting a convergent inflammatory state in macrophages as inflammation progresses. Blocking inflammasome and pyroptosis by inhibition of the inflammatory cascade by caspase-1 attenuated this convergent inflammatory state and lung pathology. The effects of caspase-1 inhibition extended beyond infected macrophages to infiltrating macrophages that are not infected with virus and resulted in reduced levels of TNF. Nonetheless, this inhibition yielded substantially increased virus production. It should be noted that it is not clear to what degree these macrophages contribute, if at all, to high titers of virus production compared with the permissive epithelial cells- although fluorescent levels of mNG virus RNA was similar in the two cell types.

Although viral uptake and the subsequent antiviral immune response, such as CXCL10, IL-18, IL-1RA production, is enhanced in presence of monoclonal antibodies, the outcome of this enhancement does not appear to be pathological when given early or late. This is in line with extensive clinical findings that show patients given convalescent plasma or monoclonal antibodies responded well to therapy and did not present with disease enhancement[83]. Several lines of evidence also suggest that FcRs are essential for antibody mediated protection and therapy[84,85]. A possible explanation for this conflicting role of antiviral antibodies is potentially explainable by the fact that the antibodies enhance infection of macrophages and thus inflammation but at the same time they neutralize virus and thereby attenuate disease leading to a net null effect consistent with the enhancing role of antiviral Abs on macrophage infection.

Viral sensing by NLRP3 in infected human macrophages

Viral RNA and particles can be detected by a variety of innate immune sensors. Among these, myeloid cell expressed inflammasomes including NLRP3, and NLRP1 can be activated by RNA viruses[55,56,86]. Human lung monocytes and macrophages in infected MISTRG6-hACE2 mice express low levels of inflammasome sensors, NLRP3, and NLRP1. Of these NLRP3 is both upregulated and activated by replicating SARS-CoV-2 in these macrophages. NLRP3 can be activated by a diverse, promiscuous set of stimuli but initially requires a priming event, which results in the transcriptional induction of NLRP3 and triggers post-translation modifications. In line with this priming step, NLRP3 transcript expression in lung tissues of infected MISTRG6-hACE2 mice was upregulated in response to infection, but interestingly was inhibited by combined therapy of anti-IFNAR and Remdesivir or by dexamethasone, all of which reduce both levels of replicating virus and inflammatory ligand. Activation of NLRP3 by some RNA viruses relies on viral replication and direct sensing of viral RNA or via other viral RNA sensors as MDA5 and RIG-I. Viral replication in the context of an early inefficient IFN response (which SARS-CoV-2 is thought to accomplish[51,53,87]), is likely a stimulus for NLRP3 activation in human macrophages. Loss of IL-18 and IL-1β production upon inhibition of viral replication in our studies strongly suggest viral replication is involved. Recent reports have also identified a possible role for NLRP3 driven inflammasome activation in infected monocytes and macrophages in post-mortem tissue samples and peripheral blood mononuclear cells (PBMC) of COVID-19 patients. Although there have been many candidates for NLRP3 activation ligands (lytic cell death upon infection, N protein[59], Orf3a[60]), the exact mechanism of NLRP3 activation is still poorly understood, especially given the diverse set of stimuli that can activate NLRP3. Nonetheless activation of other NLRs may contribute to the process, as inhibition of caspase-1 gave in general stronger inhibition of responses than inhibition of NLRP3.

Infiltrating macrophages and the essential role of viral RNA-dependent type I IFN response in disease pathology

Infection causes human macrophages to preferentially produce CXCL10 which likely attract blood monocytes to the lung where they differentiate to inflammatory macrophages. These monocytes and monocyte-derived macrophages (MDM) eventually outnumber tissue-resident macrophages; they express higher levels of TLRs and may play a central role in viral RNA detection, possibly also released by pyroptosis of infected macrophages, and the ensuing inflammatory and IFN response. IFN production is critical for the antiviral response of the early phase of disease, as also evidenced in our model by drastically higher viral loads and precipitous decline in health when the antiviral response is disabled too early by dexamethasone treatment at the peak of infection[19]. However, this same response when persistent can be pathogenic. We found that targeting either chronic viral replication or the late IFN response therapeutically *in vivo* attenuates many aspects of the overactive immune-inflammatory response, especially the inflammatory macrophage response.

Implications of our findings

Inhibition of viral replication, viral uptake and inflammasome activation in infected macrophages reduced lung hyperinflammation with high levels of inflammasome-induced cytokine IL-18, IL-1RA, and CXCL10 in infected MISTRG6-hACE2 mice. Patients with severe COVID-19 also have higher levels of IL-18, IL-1β, IL1-RA and CXCL10 [2-6,14,34,79]. Inhibition of both caspase-1 and NLRP3 resolved lung immunopathology associated with chronic disease in MISTRG6-hACE2 mice. Given that multiple reports in patient samples also identify a role for inflammasome driven hyperinflammation in pathophysiology of COVID-19, targeting inflammasome sensors or downstream effector molecules in patients may provide alternative therapeutic options for resolving chronicity in COVID-19. However, the increased virus production seen upon inflammasome blockade could pose a significant risk to the benefit of wholesale inhibition of the pathway. The combination of Remdesivir and anti-IFNAR2 antibodies could be an effective therapy for chronic COVID-19 which spares the antiviral T cell response unlike dexamethasone. More generally, the findings from our study and its implications provide alternative therapeutic avenues to be explored in the clinic and may guide novel therapeutic developments and prompt clinical trials to investigate combinatorial therapies that target viral RNA, inflammasome activation or its products and sustained IFN response.

Supplementary Tables

Table S1: Human genes that are differentially regulated in lungs of infected MISTRG6-hACE2 in response to therapeutics.

Genes that are upregulated in response to infection and downregulated in response to therapeutics (dexamethasone, anti-IFNAR+ Remdesivir) in these infected mice at 14dpi were included in the analysis (matched to Fig 1d). Normalized expression of duplicates. N=2 biologically independent mice examined over 2 -independent experiments. Differential expression analysis was performed with DESeq2 and statistical significance was deemed using Wald test.

Table S2: Cluster identifying markers and markers that identify temporal transcriptional changes associated with monocytes and macrophages in infected (4, 14 or 28dpi) or uninfected lungs of MISTRG6-hACE2 mice (matched to Fig 1g).

N=2 biologically independent mice for each condition was pooled. Marker genes for each cluster of cells were identified using the Wilcoxon test with Seurat. For the adjusted P-values the Bonferroni correction was used.

Table S3: Expression of human genes that are enriched in macrophages (clusters identified as part of Fig. 1g) during SARS-Cov-2 infection and their response to anti-IFNAR2 and Remdesivir therapy (matched to Fig. 1h). Normalized expression of duplicates analyzed.

N=2 biologically independent mice examined over 2 independent experiments. Differential expression analysis was performed with DESeq2 and statistical significance was deemed using Wald test.

Table S4: Pearson and spearman correlation values calculated for each gene for its correlation with CXCL10, TNF or TLR7 in human monocytes and macrophages at 4dpi (based on Fig. 1g, matched to Fig. 3d).

For Pearson's test, significance was based on the t-test with statistic based on Pearson's product-moment correlation coefficient cor(x, y) and following a t distribution with length(x)-2 degrees of freedom. For Spearman's test, p-values are computed using algorithm AS 89 with exact = TRUE. Correlation values, p-values (two-tailed) and FDR-adjusted p-value are presented.

Table S5: Patient specimens used for immunofluorescent (IF) staining.

Details of patient demographics for specimens use in IF staining: Age, gender, medication, time of death post-symptom onset (dps), co-morbidities, cause of death and histopathological findings. This table is presented as part of supplementary methods.

Supplementary References:

77 Zhou, Z. *et al.* Structural insight reveals SARS-CoV-2 ORF7a as an immunomodulating factor for human CD14(+) monocytes. *iScience* **24**, 102187, doi:10.1016/j.isci.2021.102187 (2021).

78 Merad, M. & Martin, J. C. Author Correction: Pathological inflammation in patients with COVID-19: a key role for monocytes and macrophages. *Nat Rev Immunol* **20**, 448, doi:10.1038/s41577-020-0353-y (2020).

79 Yang, Y. *et al.* Plasma IP-10 and MCP-3 levels are highly associated with disease severity and predict the progression of COVID-19. *J Allergy Clin Immunol* **146**, 119-127 e114, doi:10.1016/j.jaci.2020.04.027 (2020).

80 Cheung, C. Y. *et al.* Cytokine responses in severe acute respiratory syndrome coronavirus-infected macrophages in vitro: possible relevance to pathogenesis. *J Virol* **79**, 7819-7826, doi:10.1128/JVI.79.12.7819-7826.2005 (2005).

81 Hui, K. P. Y. *et al.* Tropism, replication competence, and innate immune responses of the coronavirus SARS-CoV-2 in human respiratory tract and conjunctiva: an analysis in ex-vivo and in-vitro cultures. *Lancet Respir Med* **8**, 687-695, doi:10.1016/S2213-2600(20)30193-4 (2020).

82 Zheng, J. *et al.* Severe Acute Respiratory Syndrome Coronavirus 2-Induced Immune Activation and Death of Monocyte-Derived Human Macrophages and Dendritic Cells. *J Infect Dis* **223**, 785-795, doi:10.1093/infdis/jiaa753 (2021).

83 Taylor, P. C. *et al.* Neutralizing monoclonal antibodies for treatment of COVID-19. *Nat Rev Immunol* **21**, 382-393, doi:10.1038/s41577-021-00542-x (2021).

84 Winkler, E. S. *et al.* Human neutralizing antibodies against SARS-CoV-2 require intact Fc effector functions for optimal therapeutic protection. *Cell* **184**, 1804-1820 e1816, doi:10.1016/j.cell.2021.02.026 (2021).

85 Schafer, A. *et al.* Antibody potency, effector function, and combinations in protection and therapy for SARS-CoV-2 infection in vivo. *J Exp Med* **218**, doi:10.1084/jem.20201993 (2021).

86 Bauernfried, S., Scherr, M. J., Pichlmair, A., Duderstadt, K. E. & Hornung, V. Human NLRP1 is a sensor for double-stranded RNA. *Science* **371**, doi:10.1126/science.abd0811 (2021).

87 Miorin, L. *et al.* SARS-CoV-2 Orf6 hijacks Nup98 to block STAT nuclear import and antagonize interferon signaling. *Proc Natl Acad Sci U S A* **117**, 28344-28354, doi:10.1073/pnas.2016650117 (2020).
